# Estimating and learning personality traits of and from women with borderline personality disorder

**DOI:** 10.1186/s40479-025-00320-4

**Published:** 2026-02-09

**Authors:** Lisa M. Doppelhofer, Raphael Perla, Koen M. M. Frolichs, Gabriela Rosenblau, Sabine C. Herpertz, Christoph W. Korn

**Affiliations:** 1 Department of General Psychiatry, Center for Psychosocial Medicine, University of Heidelberg, Heidelberg, Germany; 2 German Center for Mental Health (DZPG), partner site Mannheim/Heidelberg/Ulm, Heidelberg, Germany; 3 Section Social Neuroscience, Department of General Adult Psychiatry, Heidelberg University Hospital, Voßstraße 4, Heidelberg, 69115 Germany; 4 Department of Psychological and Brain Sciences, The George Washington University, Washington, D.C USA; 5 Autism and Neurodevelopmental Disorders Institute, The George Washington University, Washington, D.C USA

**Keywords:** Borderline personality disorder, Personality traits, Reinforcement learning, Computational modelling

## Abstract

**Background:**

Accurately perceiving and learning about others’ personalities is crucial for successful social relationships. Borderline Personality Disorder (BPD) is marked by unstable interpersonal dynamics, an unstable and negative self-concept, and a tendency to evaluate others unfavorably. Individuals with BPD are also more likely to be viewed negatively by others and experience social stigmatization. This study investigated whether women with BPD exhibit negativity biases when evaluating their own and others’ personality traits, and how these biases influence learning about others.

**Methods:**

Thirty women with BPD and thirty-one age- and intelligence-matched controls estimated and learned the personality of six individuals (learning profiles) by predicting their self-ratings on 40 personality traits, balanced across the Big Five personality dimensions. After each prediction, participants received feedback with the target’s actual rating, allowing participants to gradually learn each profile’s personality. Three profiles reflected BPD group personality patterns and three profiles reflected control group patterns. Crucially, participants were told that these profiles were from real individuals but were unaware of their clinical status.

**Results:**

As hypothesized, the BPD group rated themselves more negatively than controls, both at the trait level and on standardized personality measures (NEO-FFI, PID-5-BF). When evaluating others, both groups rated BPD profiles similarly, but the control group rated control profiles more favorably. For both groups, accuracy improved slightly over time for control profiles but not BPD profiles, suggesting that BPD trait patterns are inherently harder to learn. Computational modeling indicated that both groups used fine-grained learning strategies—regardless of profile type—with no credible group differences in learning mechanisms.

**Conclusion:**

These findings demonstrate that individuals with BPD exhibit pervasive negativity biases in self- and other-evaluations, yet retain intact social learning capacities. This suggests that interpersonal difficulties in BPD may stem more from negatively biased expectations than from deficits in learning. Moreover, the inherent difficulty in learning about BPD-like personality profiles—observed in both groups—may hinder mutual understanding and contribute to persistent social challenges. Importantly, the intact learning capacity points to a valuable therapeutic resource: targeting negative social expectations may help to reduce bias and improve social functioning in BPD.

**Supplementary Information:**

The online version contains supplementary material available at 10.1186/s40479-025-00320-4.

## Introduction

People differ widely in their personalities, which makes the ability to estimate and learn others’ personality traits crucial for navigating social interactions and relationships. Personality traits are commonly defined as consistent patterns of thoughts, feelings, and behaviors that remain relatively stable over time and across different situations [1]. Traits of others are typically inferred through repeated interactions and observation. But impressions can also be formed rapidly and spontaneously, often based on minimal information and even without direct contact [2–8].

The process of evaluating and learning others’ personality traits is closely intertwined with individuals’ self-views [9–12]. Importantly, negative self-views combined with maladaptive social estimation and learning processes are thought to contribute to interpersonal dysfunction across various psychiatric disorders, including Borderline Personality Disorder (BPD) [13–18]. BPD is characterized by impulsivity, intense emotional instability, and difficulties in interpersonal relationships [19, 20]. These social difficulties often manifest as fewer, more unstable, and ambivalent interpersonal connections [21–24]. In addition, people with BPD frequently experience stigmatization—not only from the general public but also within the healthcare system [25–28]. One potential contributor to these interpersonal challenges may be atypical processes in how individuals with BPD evaluate and learn about others’ personalities, as well as how others evaluate and learn about them. However, empirical and analytical approaches to investigate such maladaptive social learning mechanisms are surprisingly scarce and heterogeneous. In the present study, we provide a model-based framework to test how women with BPD perceive themselves and how they learn about the personalities of individuals with and without BPD. This framework also allowed us to examine how nonclinical control participants learn about the personalities of individuals with and without BPD.

While nonclinical populations typically maintain positive self-perceptions and self-related positivity biases [29–32], individuals with BPD tend to hold less positive, more negative, and more inconsistent self-views [14, 33–35]. This pattern is also reflected in self-report measures of the Big Five personality dimensions [36, 37]: patients with BPD score higher on Neuroticism and lower on socially desirable dimensions such as Agreeableness and Conscientiousness compared to controls [38, 39]. Similarly, on questionnaires assessing maladaptive personality trait domains, individuals with BPD have been shown to score higher across all domains than healthy controls [40] and clinical samples with other personality disorders [41]. Compared to controls, individuals with BPD focus more on negative self-relevant information and are particularly prone to internalize negative feedback from others [14, 33]. Importantly, individuals with BPD are also perceived more negatively by others. After a brief interaction, healthy controls tend to rate the personality traits of people with BPD more negatively than those of other healthy controls [14]. Individuals with BPD are rated higher in depression, emotional instability, and anxiety based on photographs compared to healthy controls [42], and are judged as less trustworthy, and less likeable based on short video clips [27, 43, 44].

Further evidence suggests that individuals with BPD struggle to recognize positive social signals and show hypersensitivity to negative social signals [20, 45]. In facial emotion recognition tasks, they exhibit a heightened sensitivity to threat-related cues [45–47] and deficits in recognizing neutral and positive facial expressions [48–50]. When evaluating others, they tend to view them as more negative, inconsistent, and aggressive compared to controls [14, 43, 51–53]. They also anticipate greater selfishness [54] and lower trustworthiness in others [55]. While previous studies have highlighted negativity biases in individuals with BPD, it remains unclear whether social estimation and learning processes are biased—and how such biases can be modelled.

Adaptive social estimation and learning require flexible representations and continuous adjustments of complex social knowledge structures [16]. Computational modeling offers a powerful approach for advancing research on these processes because it requires researchers to formalize theories, specify mechanisms, and derive precise predictions that can be explicitly tested [56, 57]. In this way, computational models move beyond descriptive accounts by providing explanatory, mechanistic frameworks with psychologically interpretable parameters [56, 58, 59].

Within this framework, reinforcement learning (RL; [60]) models have emerged as a particularly robust and parsimonious class of models for describing how humans learn from trial and error. In RL algorithms, learning is defined as a stepwise reduction of prediction errors (PEs)—the difference between an initial estimate (or prediction) and the subsequent feedback (or outcome). These PEs are used to step-by-step update future predictions. Crucially, these error signals correspond closely to neural responses in reward-related brain circuits [61–65], providing strong evidence for the validity and importance of RL theory as a mechanistic account of human learning. RL theory has proven to be a powerful framework in explaining a wide range of cognitive and social tasks [59, 60, 66–71], and has increasingly been applied to elucidate key aspects of psychiatric disorders [72–79]. Previous studies have demonstrated that RL captures how humans learn about others’ intentions [80], emotions [81], trustworthiness [82], and preferences [83]. Moreover, combining RL algorithms with conceptual knowledge highlights how humans manage the complexity of social environments: rather than relying solely on costly trial-and-error computations, people draw on abstract representations and prior expertise to generalize flexibly while minimizing cognitive effort [84].

Our own work has indicated that, in addition to RL, at least two additional concepts play an important role when learning about others’ traits or preferences: reference points and granularity [85]. Reference points capture prior knowledge or assumptions about average members of specific groups and serve as a baseline when encountering new individuals. For example, when meeting a medical student for the first time, people may draw on their knowledge of the “average medical student” to generate initial predictions about that person’s personality. Granularity, in turn, refers to the level of detail that people apply when updating these predictions after receiving new information. In our framework, updates are guided by the Big Five personality factors [36, 37]. A coarse-grained representation updates only at the level of broad factors. For instance, if the medical student is observed to be very organized, this information would raise the estimate of their overall Conscientiousness, which in turn increases expectations for all traits within that factor (e.g. reliability, diligence) equally. A fine-grained representation updates predictions at the level of individual traits, weighted by their similarity to the newly observed trait. In the same example, learning that the student is organized would strongly increase expectations about closely related traits such as diligence or reliability. It would also influence more distant traits, such as empathy from the Agreeableness factor, though to a much smaller extent. While the fine-grained model allows for more nuanced and flexible generalizations, it is also more cognitively complex. The coarse-grained model, in contrast, is more parsimonious and resource-efficient, and may be particularly adaptive when information about a new person is sparse or when only broad impressions are needed [85]. Recent studies confirm that people indeed exhibit conceptual trait knowledge, actively use it when making social judgments, and that these knowledge structures have neural correlates [86, 87]. Building on this foundation, our own work using computational models incorporating these two concepts, has shown that the learning behavior of non-clinical adults was best described by reference points and levels of generalization that were appropriately adjusted when learning about others [85]. We proposed that such models may also help identify impairments in social learning in clinical populations, which may stem from insufficiently adjusted reference points or generalizations when forming personality inferences [16]. Supporting this view, a previous study has shown that adolescents with autism spectrum disorder relied on their own preferences as reference points and showed reduced updating during the learning process [83].

Here, we use this modeling framework to test whether individuals with BPD show characteristic restrictions and negative biases in social learning. We adapted a social learning task from our previous studies [14, 31, 83, 85, 88] to investigate potential differences between individuals with BPD and controls in estimating and learning others’ personality traits. Specifically, we examined how women with and without BPD (i.e., controls; CON) learned about the personality traits of both BPD and control groups. We proposed that individuals with BPD, would rely on their (negative) self-views as reference points and on overgeneralized coarse-grained representations [16]. In addition to reference points and granularity, we test extended model variants incorporate hierarchical estimation to recover the initial expectation for each trait prior to any learning (referred to as V0 estimates; see Methods section and Results section). These parameters capture participants’ prior beliefs about average trait expressions and are estimated from the observed learning dynamics rather than specified a priori.

This study was preregistered, and we briefly summarize the preregistered hypotheses (https://osf.io/jwky9). The hypotheses are numbered [H1] to [H6] for easier reference throughout the article. In the methods section, we provide more technical details on these hypotheses. We predicted that individuals with BPD would rate themselves [H1] more negatively on trait adjectives and personality questionnaires (NEO-FFI, [36, 89]; PID-5-BF, [90–92]) than controls. In addition, we expected that the BPD group compared to the control group would also rate others more negatively on trait adjectives [H2]. Furthermore, we anticipated that controls would more accurately estimate the personality profiles of other controls compared to individuals with BPD [H3.1], and that individuals with BPD would demonstrate greater accuracy when estimating the profiles of other BPD participants than those of control participants [H3.2]. Based on our social learning framework [16], we hypothesized that the two groups would differ in the cognitive strategies during trait learning: we expected individuals with BPD to rely more on their self-ratings and coarse trait similarities, and controls to use average population ratings and fine-grained item-level similarities—this pattern holding true regardless of the profile being evaluated [H4]. Additionally, we planned to test if individuals with BPD would exhibit a lower overall learning rate compared to controls [H5] and if they would learn more from negative PEs than from positive ones [H6].

## Methods

### Study procedures

This study is part of a broader project investigating social decision making in women with BPD, including additional behavioral and fMRI experiments, which are not part of the scope of the current study (see [93] for a published article on social decision-making in women with BPD in an overlapping sample of participants). The project received approval from the local ethics committee of the Faculty of Medicine at Heidelberg University (S-137/2020). The specific rationale, hypotheses and approach of this study were preregistered on the Open Science Framework on May 30, 2022 (https://osf.io/jwky9) prior to data analysis. Any protocol modifications are detailed in the methods section.

Data collection took place between October 2021 and May 2023. Participants in the control group were age-matched during the recruitment. A brief intelligence screening confirmed comparable cognitive skills between the groups (see Participants section). Participants were recruited in Heidelberg and nearby towns. We additionally recruited inpatients at the Department of General Psychiatry, University of Heidelberg. Participants in the control group received a base rate of €50 and those with BPD received a base rate of €60 (to account for the extended diagnostic interview time) for completing all experiments of the larger project. Following a telephone prescreening, eligible participants were invited to the study. During the in-person assessment, participants took part in a diagnostic interview, a brief cognitive task (see Clinical assessment section), and a series of self-report questionnaires using the German online survey platform SoSci Survey [94] (see Supplementary Material). They completed the behavioral tasks of the current study and unrelated behavioral experiments. Prior to participation, they were informed about the study procedures and provided written consent. All participants were fully debriefed at the end of the study.

### Participants

#### Clinical assessments

We used two standardized diagnostic instruments: the Mini-DIPS Open Access [95], a shortened version of the Diagnostic Interview for Mental Disorders, to assess Axis I disorders; and the German version of the International Personality Disorder Examination (IPDE; [96, 97]) to evaluate BPD criteria. To be included in the BPD group, participants had to meet at least four IPDE criteria, allowing for the inclusion of sub-threshold cases. Control participants could meet no more than one IPDE criterion (see Supplementary Material for details). Additionally, all participants completed the mini-q [98], a three-minute speeded reasoning test, to ensure that cognitive skills are matched between the groups. All clinical interviews were conducted by trained raters, with typical sessions lasting approximately three hours.

#### Sample description

We analyzed all valid datasets for the current study and included 30 individuals with BPD and 31 controls (see Supplementary Material for inclusion and exclusion procedure). The control group was matched for age and cognitive abilities as measured with the mini-q (see Table 1). Participants in the BPD group differed significantly from the control group in their level of education, professional qualification, and employment status (see Table 2). The BPD group consisted of 30 participants, of whom 29 had been formally diagnosed with BPD prior to study enrollment. One participant had only a provisional BPD diagnosis from their psychotherapist, which was confirmed through the IPDE interview. Consistent with the heterogeneity of BPD, participants in the BPD group met diagnostic criteria through different combinations of symptoms in the IPDE. Importantly 73.3% endorsed the criterion of unstable and intense interpersonal relationship, confirming that interpersonal difficulties were present in the majority of cases. In addition, several other interpersonal or identity-related criteria (e.g., affective instability, identity disturbance, efforts to avoid abandonment) were endorsed by more than half of the sample, further underscoring the relevance of the current paradigm for studying social learning processes (the full distribution is provided in the Supplementary Material). In the BPD group, 86.67% of participants reported symptoms of comorbid diagnoses according to the Mini-DIPS interview (see Supplementary Material Table S1), most commonly recurrent major depressive disorder (50%), social anxiety disorder (43.33%), and post-traumatic stress disorder (33.33%). Approximately half of the BPD participants (53.33%) were taking psychotropic medication, primarily antidepressants (see Supplementary Material Table S2). Medication intake information was self-reported by participants.

**Table 1:** Age and mini-q summary table

	BPD		CON		Welch’s t-test						Wilcoxon rank sum test		
	*M*	*SD*	*M*	*SD*	*t*	*DF*	*CI (LL)*	*CI (UL)*	*p*	*d*	*z*	*p*	*r*
Age	26.07	5.72	26.00	5.14	−0.048	57.873	−2.856	2.722	0.962	−0.012	0.196	0.845	0.025
Mini-q scores	34.30	12.18	34.03	8.20	0.100	50.613	−5.087	5.622	0.920	0.026	*-*0.094	0.925	0.012

**Table 2:** Demographic characteristics of both groups

	BPD (N = 30)		Control (N = 31)		*p*
	*N*	%	*N*	%	
Education					
Secondary general school (9 years)	1	3.33	0	0	**< 0.005**
Intermediate school (10 years)	6	20	0	0	
High school diploma (13 years)	23	76.67	31	100	
Professional qualification					
Non, in professional training	7	23.33	15	48.39	**< 0.001**
Non, not in professional training	4	13.33	0	0	
Apprenticeship	5	16.67	0	0	
Vocational school	9	30	2	6.45	
Technical school	0	0	1	3.23	
University	5	16.67	13	41.94	
Employment status					
Student	5	16.67	21	67.74	**< 0.001**
In training	5	16.67	0	0	
Employed	10	33.33	8	25.81	
Unemployed	4	13.33	1	3.23	
Retired or homemaker	2	6.67	0	0	
Other	4	13.33	1	3.23	

*p* = *p*-values from Fisher’s exact test for count data. Significant results in bold font

### Experimental procedure

We assessed participants’ dimensional personality structure to test if participants in the BPD group evaluated themselves more negatively than participants in the control group. To this end, we assessed the Big Five personality dimensions (Neuroticism, Extraversion, Openness, Agreeableness, Conscientiousness) with the NEO Five-Factor Inventory (NEO-FFI; [36, 89]). Additionally, we characterized the presence and frequency of five maladaptive personality domains (Negative Affect, Detachment, Antagonism, Disinhibition, Psychoticism) with the Personality Inventory for DSM-5–Brief Form (PID-5-BF; [90–92]).

**Fig. 1:**
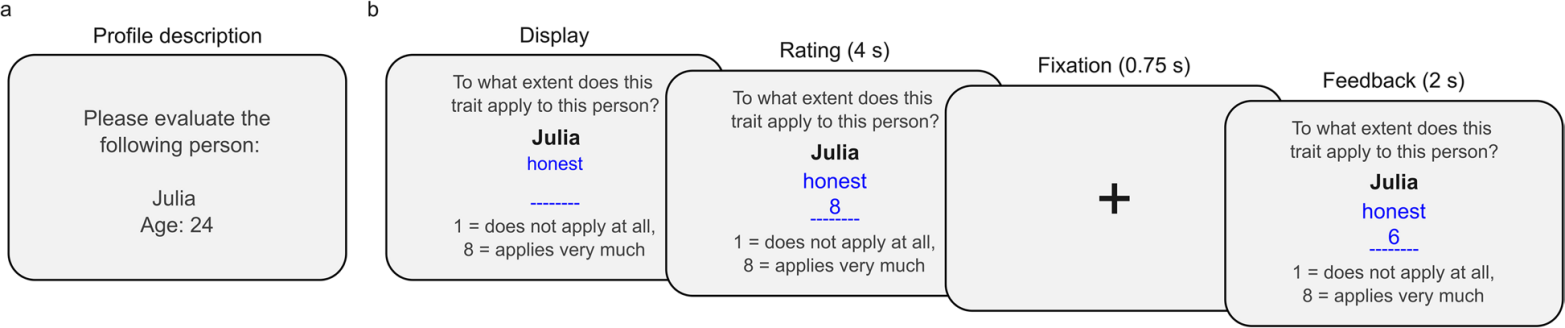
Personality learning task procedure. **a** Example of a profile description presented at the beginning of a learning block, showing the person’s (profile’s) name and age. **b** Structure of a single trial within the learning block. On each trial, participants estimated the extent to which a trait word applied to a given person. After providing their rating (4 s), a fixation cross (0.75 s) appeared, followed by feedback (2 s) showing the profile’s self-rating on the same trait word. This feedback allowed participants to gradually learn about the person and adjust their estimates for the following traits accordingly. A second fixation (0.75 s) followed the feedback, after which the next trait word was presented (not depicted). Each profile was evaluated on 40 trait words, with a total of six profiles presented (three BPD profiles, three control profiles). Profiles only differed in their names, ages and self-ratings (three reflecting BPD personality patterns and three reflecting control group personality patterns); they did not differ visually. Importantly, participants were not informed that the profiles represented either BPD or control individuals

**Fig. 2:**
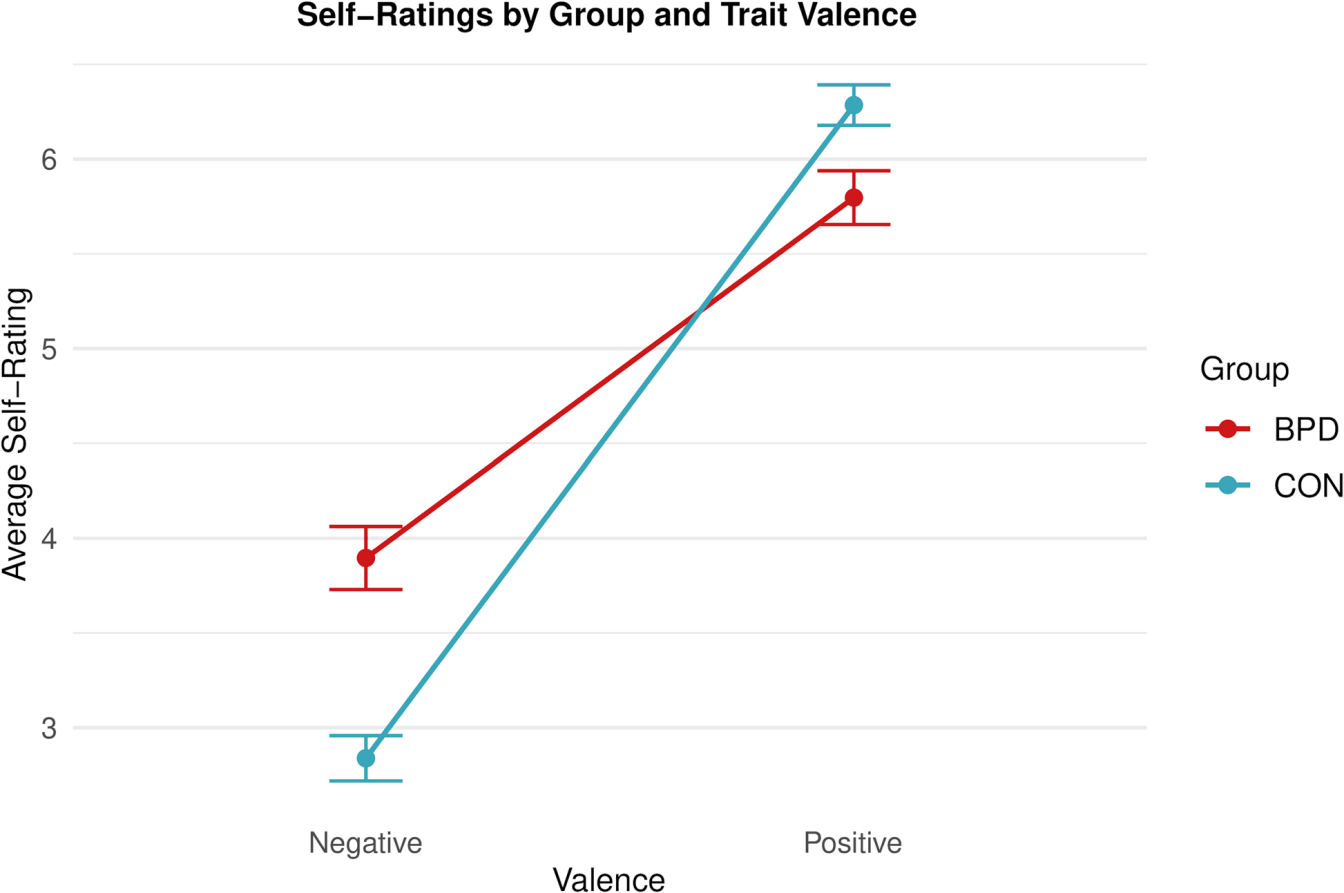
Self-ratings on positive and negative trait words in the BPD group (red) and control group (blue). The graph indicates that the BPD group gave more unfavorable self-ratings, that is higher ratings on negative traits and lower ratings on positive traits (significant Group x Valence interaction in an LME model). Lines show observed means, error bars indicate 95% confidence intervals based on a normal distribution. Ratings on negative items are not reverse coded

The behavioral task consisted of two parts: 1) estimating and learning about the personality traits of six individuals, which we call “learning profiles,” (Fig. 1) and 2) providing self-ratings on the same traits. During the first part, the learning phase, participants were shown six personality profiles. Each profile consisted of self-ratings on 40 personality traits. These 40 traits were selected to reflect items found in the Big Five personality model, with 20 positive and 20 negative traits (e.g., honest and anxious). Between 7 and 9 traits were shown per Big Five factor (see Supplementary Material Table S3 for the full list of original German and translated English trait words). Each profile was paired with a randomly assigned common German female name and an age within the study sample’s range (see Supplementary Material for the specific names and ages).

The three BPD profiles were drawn from self-ratings of participants with BPD from a previous study [14]. These profiles were selected from participants whose trait ratings closely matched their group averages—specifically, within ± 1 SD on most traits (a maximum of six traits exceeded this range). Due to a deviation from the intended implementation, the control profiles were not selected based on the ± 1 SD criterion. These profiles remained consistent with plausible control group patterns, although they did not strictly adhere to the preregistered selection parameters.

Participants were informed that the profiles represented real individuals from a previous study, but they were not told whether profiles came from a control group or a BPD group. Each profile learning block started with a brief description including the name and age of the person. Participants rated each profile’s traits on an 8-point scale (1 = does not apply at all, 8 = applies very much), learning about one profile at a time. For each trial, one trait word (e.g., “honest”) was displayed on the screen, and participants had 4 s to estimate the profile’s self-rating. Following each estimate, 2 s of feedback were shown with the veridical rating from the person in question, allowing participants to learn about the profile’s personality traits over time. Each profile learning block consisted of 40 trials (40 different traits), and all participants evaluated all six profiles (240 trials in total). In the second part of the task, participants rated themselves on the same 40 traits. The on-screen instructions are provided in the Supplementary Material.

The order of the trait words was randomized for each profile. The order of the profiles was CON, BPD, CON, BPD, BPD, CON (except for three participants). The order of the self-ratings was not randomized (similar to many questionnaires) to avoid inter-individual differences that are solely due to the order in which items are presented.

### Analyses

#### Task specific exclusion procedures

As preregistered, we excluded trials with trait words (items) that participants did not understand. Participants could mark these words afterwards on a printout with all words. Eleven participants with BPD and 12 controls marked one or two items. That is, for each of these participants a maximum of two items were excluded. Additionally, for two participants in the BPD group, we chose to exclude one profile each, as they reported that the profile’s name influenced their responses (this exclusion criterion was not preregistered).

#### Data preprocessing

We reverse-coded responses on negative items/traits for all PE, self-rating, and other-rating analyses that did not include item valence (e.g., a rating of ‘1’ for a negative trait was transformed into a positive trait with a recalculated rating of ‘9 − 1 = 8’).

#### Regression analyses

We used linear mixed effects (LME) models to test the preregistered hypotheses [H1] to [H3.2]. We hypothesized that individuals with BPD would rate themselves more negatively on personality traits and personality questionnaires than controls (NEO-FFI, PID-5-BF) [H1]. Additionally, we expected that individuals with BPD would rate others more negatively on personality traits than controls. That is, the BPD group’s estimates of others’ personality traits would be numerically smaller for positive trait words and larger for negative trait words compared with the control group’s estimates [H2]. The study employed a mixed 2 × 2 design, with two groups (BPD/CON) and two types of learning profiles (BPD/CON). We hypothesized an interaction effect for absolute PEs, with specific expectations that control participants’ estimates for control profiles would be closer to the true values (smaller PEs) than BPD participants’ estimates [H3.1], and that BPD participants’ estimates would be closer to the true values (smaller PEs) when learning about other BPD participants compared to control profiles [H3.2].

We used the standard alpha *p* < 0.05 criterion for determining significant results, and hence rejecting the null hypotheses. All analyses were conducted in R. Linear mixed effects (LME) models were fitted with the package *lmerTest* [99] by restricted maximum likelihood (REML). We used the *tab_model* function from the *sjPlot* package [100] to report summary tables from the LME models with the Satterthwaite’s approximation for computing degrees of freedom for *p*-values, standard errors and confidence intervals (CI). For all LME models we used the BPD group and the BPD profile type as reference categories. We calculated Type III analysis-of-variance (ANOVA) tables for the LME models with Satterwaite’s method using the *anova* function in R. Note, that under treatment coding, main effects in the presence of interactions are interpreted relative to the reference levels of the other factors. Post-hoc comparisons were conducted by calculating estimated marginal means (EMMs) from the fitted LME model with the *emmeans* package [101]. All *p*-values for the post-hoc comparisons were adjusted with the *Tukey* method.

We report post-hoc tests for LME and ANOVA results, which reached significance at *p* < 0.05, in the results section, as these address our preregistered hypotheses. Summary tables for the LME models and the Type III ANOVAs are provided in the Supplementary Material.

##### Self-ratings on trait adjectives

To test if individuals in the BPD group rate themselves more negatively than individuals in the control group [H1], we fitted an LME model and examined the effect of *Group* (BPD/CON) on the self-ratings. The model included *Group* as a fixed effect and random intercepts for both the 61 subjects and the 40 items. Ratings on negative items were reverse coded.

LME model formula: 

$$\begin {aligned} \textrm{Self-ratings} &\sim\textrm{Group} + (1 | \textrm{ Subject}) \\ &+ (1 | \textrm{ Item})\end {aligned}$$

 

In addition, we explored potential group differences between positive and negative items. We tested *Group* (BPD/CON) and *Valence* (positive/negative) and their interaction as fixed effects and random intercepts for subjects and items. Here, we explicitly contrasted the positive versus negative valence of the items (hence did not reverse code ratings on negative items). To investigate whether self-ratings differ between groups for positive or negative items, post-hoc comparisons of EMMs were calculated. This approach estimated the mean response for each *Group* within each level of *Valence*.

LME model formula: 

$$\begin {aligned} \textrm{Self-ratings} &\sim\textrm{Group} * \textrm{Valence}\\ &+ (1 | \textrm{ Subject}) + (1 | \textrm{ Item})\end {aligned}$$

 

##### Self-ratings on personality inventories

To test whether the BPD group exhibited more unfavorable self-ratings on the Big Five [36, 37] and maladaptive personality traits [H1], as measured by the NEO-FFI [36, 89] and the PID-5-BF [90–92], we conducted MANOVAs and subsequent post-hoc t-tests. For the MANOVAs, Pillai’s trace was computed. Welch’s t-tests were performed, and *p*-values were adjusted for multiple comparisons using the false discovery rate (FDR) method. Effect sizes (Cohen’s d) were calculated with the *effsize* [102] package.

##### Other-ratings

We hypothesized that individuals with BPD would rate others more negatively—lower on positive traits and higher on negative traits—than controls [H2]. We fitted an LME model and tested the association between ratings of others’ personality traits and the effect of *Group* (BPD/CON) and *Valence* (positive/negative), and their interaction. We tested *Group* and *Valence* and their interaction as fixed effects and random intercepts for subjects and items. Ratings on negative items were not reverse coded. To investigate whether other-ratings differ between groups for positive or negative items, we conducted pairwise comparisons.

LME model formula: 

$$\begin {aligned} \textrm{Other-ratings} &\sim\textrm{Group} * \textrm{Valence} \\ &+ (1 | \textrm{ Subject}) + (1 | \textrm{ Item}) \end {aligned}$$

To explore whether other-ratings differed between groups (BPD/CON) depending on *Profile type* (BPD/CON), we fitted an LME model and tested the effects of *Group* and *Profile type*, and their interaction on other-ratings as fixed effects and random intercepts for subjects and items. Ratings on negative items were reverse coded. We calculated pairwise comparisons for *Group* within each level of *Profile type* (BPD/CON) to examine differences between the BPD and control group.

LME model formula:

$$\begin {aligned} \textrm{Other-ratings}&\sim\textrm{Group} * \textrm{Profile type} \\ &+ (1 | \textrm{ Subject}) + (1 |\textrm{ Item}) \end {aligned}$$

 

##### Prediction errors

To calculate the PEs, we subtracted the participant’s rating/prediction (P) from the profile’s self-rating/feedback (F) for each trial (t). We used the absolute PE for all subsequent analyses.

$${\mathrm{PE}}_{\mathrm t}=\;\mid{\mathrm F}_{\mathrm t}-{\mathrm P}_{\mathrm t}\mid$$

We expected that the control group would be more accurate than the BPD group in predicting control profiles [H3.1] and that the BPD group would be more accurate in predicting BPD profiles than CON profiles [H3.2]. We set up an LME model and conducted corresponding post-hoc tests. The LME model included the effects of *Group* (BPD/CON), *Profile type* (BPD/CON), and their interaction on PEs and random intercepts for subjects and items. We conducted pairwise comparisons to investigate whether PEs differed between groups across different levels of profile types and between profiles across different levels of group.

LME model formula: 

$$\begin {aligned} \textrm{PE} &\sim \textrm{Group} * \textrm{Profile type} \\ &+ (1 |\textrm{ Subject}) + (1 |\textrm{ Item}) \end {aligned}$$

 

In addition, we set up an LME model to test if PEs decrease over time (as a linear effect of trial number), as an indication of learning. We tested the effect of *trial* and random intercepts for subjects and items. The LME model is a more accurate procedure than the initially preregistered method of using Pearson correlation, which disregards the hierarchical nature of our data, potentially resulting in inflated estimates and biased standard errors [103, 104].

LME model formula: 

$$\textrm{PE} \sim \textrm{Trial}+ (1 |\textrm{ Subject}) + (1 | \textrm{ Item})$$

 

To explore whether participants became more accurate in estimating the profiles across trials depending on the group and profile type, we set up an LME model and tested the effects of *Trial*, *Group* (BPD/CON), *Profile type* (BPD/CON), and their interactions on PEs. The model included random intercepts for subjects and items. We conducted pairwise comparisons of EMMs of linear trends to test whether the slopes differed between groups across the different levels of profiles.

LME model formula: 

$$\begin {aligned} \textrm{PE} & \sim \textrm{Trial} * \textrm{Group} * \textrm{Profile}\;\textrm{type} \\ & + (1 |\textrm{ Subject}) + (1 |\textrm{ Item}) \end {aligned}$$

 

#### Computational models

We sought to understand how participants with and without BPD learn and adjust their expectations about profiles of people with and without BPD during the task by testing eight different computational models (see model description below). Conventional tests of mean and slope differences detect group effects but leave theoretical mechanisms unexamined (see [105] for a recent treatment). In contrast, computational models embed explicit mechanistic assumptions to bridge theory and statistical inference [56, 57, 106], and yield psychologically interpretable parameters that index latent cognitive processes instead of merely summarizing group-level outcomes [107, 108]. By modeling the full data‐generation process, these approaches can distinguish between competing mechanisms that produce similar mean patterns [109, 110], and permit formal comparison of models embodying different theoretical assumptions, thereby driving systematic theory refinement [111, 112]. Motivated by these strengths, we tested eight models adapted from Frolichs et al. [85] organized into three families of generative assumptions.

Based on prior literature, we expected that individuals with BPD would rely more on self-ratings and coarse trait similarity (Model 4, see below), while controls would use population mean ratings and fine-grained similarity (Model 8, see below) [H4]. We expected these models to perform best regardless of the profiles participants learned about. Furthermore, if BPD participants’ behavior could be explained by models including learning rates from trial-by-trial prediction errors, we expected that their learning rates for others’ traits would be smaller than those of controls [H5]. Finally, if participants’ behavior was best explained by models with separate learning rates for positive and negative prediction errors, we had planned to test models with separate learning rates [H6]. However, we refrained from testing [H6] due to limitations in the task design, which we had not adequately considered beforehand.

Below, we provide a brief conceptual overview of the three classes of computational models we employ. A more detailed mathematical explanation is included in the Supplementary Material.

##### No learning models

In this category, participants are assumed not to update their beliefs over time. Instead, their responses rely on fixed reference points: either the average value observed in the overall population or the individual’s own trait expressions. These reference points act as “default predictions” and remain unchanged despite new information.

##### Coarse granularity models

These models propose that participants learn in a generalized, overarching way. Rather than tracking every individual trait, they update their overall expectations across broad personality dimensions, specifically the Big Five. When feedback differs from their expectations, participants adjust their beliefs in the direction of the feedback. The speed of this adjustment is allowed to vary across individuals. The models also account for variability in responses by incorporating a degree of randomness or noise. Within this category, one model operates without any reference points, while others use either the population average or the individual’s own trait expression as a reference point.

##### Fine granularity models

The Fine Granularity Models provide the most detailed representation of the learning process, assuming that participants maintain distinct expectations for each individual trait. These models take into account the interconnections between traits, where learning about one trait can influence expectations about related traits. To achieve this, the models incorporate a set of known relationships between traits that were established in previous research [14, 31]. Similar to the coarse granularity models, the fine granularity models include versions that balance learning with reliance on a reference point (population or self) by weighting each source of information accordingly.

##### Model fitting

We used the Stan programming language [113] and its R interface [114] to fit the models to the participants’ data. Stan was chosen primarily for its strength in hierarchical estimation, which typically provides more accurate parameter estimates than non-hierarchical methods [71, 115]. In doing so, we considerably improved on our previous model fitting approach, in which models were fitted to each participant’s data sequentially. Moreover, the Bayesian framework in Stan provides full uncertainty quantification for every parameter, facilitating direct comparisons of group-level effects (e.g., in initial expectations or learning rates) by sampling from the posterior difference-distribution of their estimated population means and examining coverage metrics such as the 95% credible interval. The technical details of our model fitting procedure, including specific algorithms and diagnostic criteria, are provided in the Supplementary Material.

#### Model comparison

Following Vehtari et al. [116], we compared the fit of our models by assessing how well they predicted data from new, unseen participants. Specifically, we employed a cross-validation approach (called leave-one-group-out-cross-validation, LOGO-CV) in which we systematically withheld one participant’s data at a time, trained the model on the remaining participants, and then assessed its ability to predict the held-out data. In other words, each participant’s data were excluded once, and the model’s predictions for that participant were compared to the observed values. To quantify predictive accuracy, we averaged the squared errors across all participants, yielding an overall mean squared error (MSE) score that reflects each model’s ability to generalize to new data.

To estimate the uncertainty in model comparisons, we calculated the standard errors of the differences between model scores. A model was considered a better fit than another if its performance difference exceeded the associated standard error by at least a factor of two. This criterion is approximately equivalent to the decision boundary for statistical significance commonly used in frequentist statistics [117]. A more detailed and mathematically thorough description of our model comparison approach is provided in the Supplementary Material.

## Results

### Regression analyses

#### Self-ratings on trait adjectives

In line with our hypothesis [H1] that individuals with BPD have more negative self-views than controls, we found that they rated themselves less favorably on the 40 personality traits. We found that the control group gave more favorable trait ratings, indicated by a significant effect of *Group* on self-ratings in the LME model (*b* = 0.77, 95% CI [0.56, 0.99], *SE* = 0.11, *t*_*(58.76)*_ = 7.16, *p* < 0.001; see Supplementary Table S4 for the model summary and Supplementary Table S5 for Type III ANOVA summary).

We additionally explored effects of trait valence (see Supplementary Tables S6-8 for full model summaries and post-hoc contrasts). Individuals with BPD rated themselves less positively on positive traits (*M*_*BPD*_ = 5.79, *SD*_*BPD*_ = 0.582, *M*_*CON*_ = 6.28, *SD*_*CON*_ = 0.351; EMMs contrast: *b* = −0.49, 95% CI [−0.71, −0.27], *SE* = 0.11, *t*_(121.67)_ = −4.44, *p* < 0.001) and more negatively on negative traits (*M*_*BPD*_ = 3.89, *SD*_*BPD*_ = 0.653, *M*_*CON*_ = 2.84, *SD*_*CON*_ = 0.576; EMMs contrast: *b* = 1.05, 95% CI [0.84, 1.27], *SE* = 0.11, *t*_(120.64)_ = 9.57, *p* < 0.001) compared to controls (Fig. 2).

#### Self-ratings on personality inventories

In line with our hypothesis [H1] that individuals with BPD rate themselves more negatively than controls on personality trait questionnaires, we found that participants with BPD rated themselves less favorably on the personality inventories NEO-FFI and PID-5-BF. We found a significant main effect of *Group* on the personality dimensions of the NEO-FFI (*V* = 0.77, *F(5,55)* = 36.1, *p* < 0.001). Post-hoc tests, corrected for multiple comparisons, revealed that individuals with BPD scored significantly higher than controls on Neuroticism, and significantly lower on Extraversion, Agreeableness, and Conscientiousness. We found no significant group difference in the Openness factor. The full MANOVA model summary and the post-hoc comparisons are reported in Supplementary Tables S9-10.

Similarly, we found a significant main effect of *Group* on the PID-5-BF domain scores (*V* = 0.68, F(5,55) = 23.86, *p* < 0.001). After correcting for multiple comparisons, individuals with BPD scored significantly higher than controls across all five maladaptive trait domains of the PID-5-BF. A detailed model summary and post-hoc results are provided in Supplementary Tables S11-12.

#### Other-ratings

In contrast to our hypothesis [H2], the BPD and control groups did not differ in how favorably they rated others across profile types in the learning task (see Supplementary Tables S13-15 for full model summaries and post-hoc contrasts). Pairwise comparisons of group differences within each level of valence revealed no significant effects. Specifically, there was no significant group difference for negative items (*M*_*BPD*_ = 3.78, *SD*_*BPD*_ = 1.48; *M*_*CON*_ = 3.64, *SD*_*CON*_ = 1.41; EMMs contrast: *b* = 0.14, *SE* = 0.07, *z* = 1.83, *p* = 0.067) or for positive items (*M*_*BPD*_ = 5.67, *SD*_*BPD*_ = 1.33; *M*_*CON*_ = 5.76, *SD*_*CON*_ = 1.27; EMMs contrast: *b* = −0.09, *SE* = 0.07, *z* = −1.22, *p* = 0.221).

**Fig. 3:**
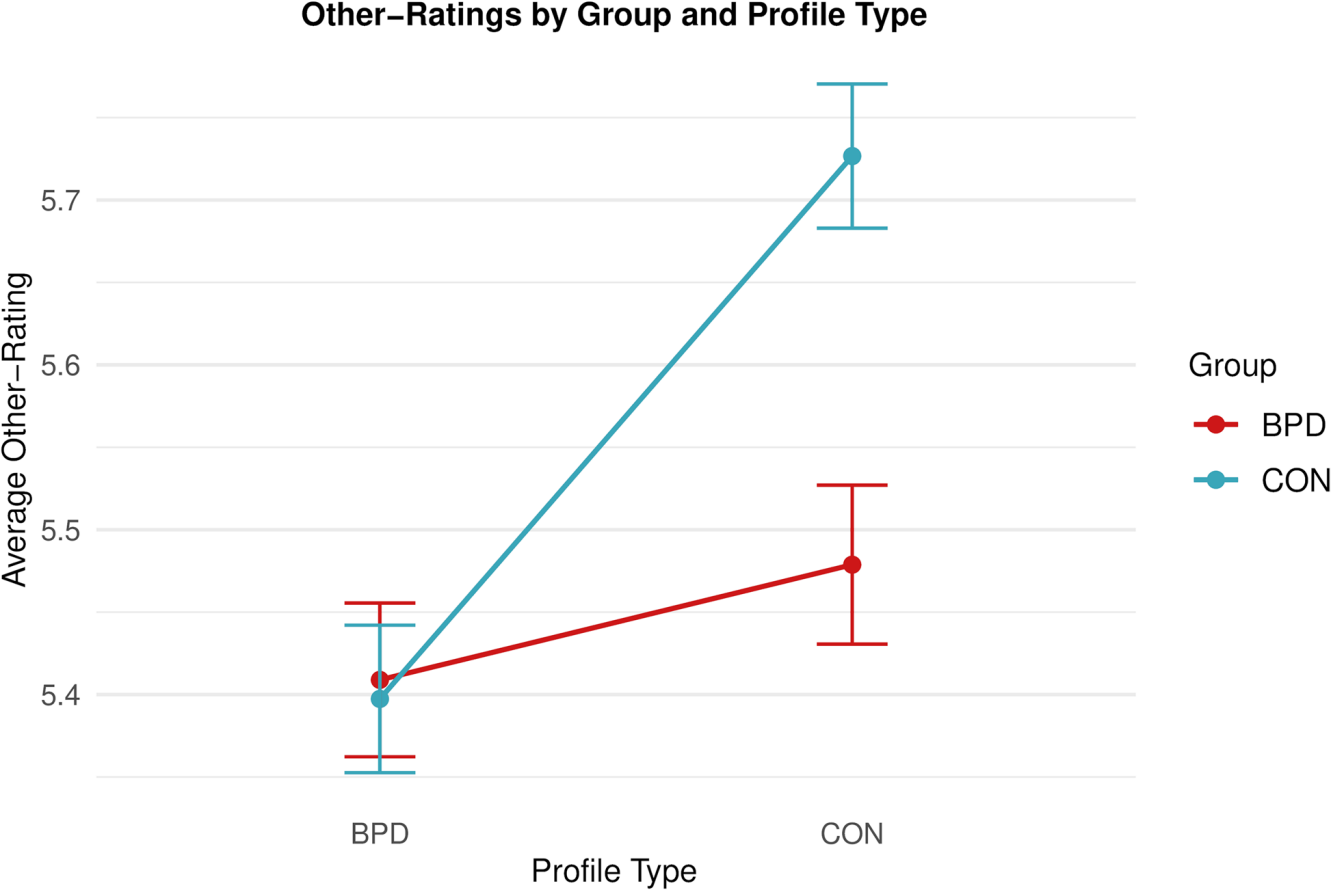
Other-ratings of BPD profiles and control profiles in the BPD group (red) and the control group (blue). The graph indicates that participants in the control group rate the control profiles most favorably (significant *Group* and *Profile Type* interaction in an LME model, and significant post-hoc contrasts for the effect of *Group* on the control profiles, but not for the BPD profiles). Lines show observed means, error bars indicate 95% confidence intervals based on a normal distribution. Ratings on negative items are reverse coded

To explore whether potential group differences were driven by a specific profile type, i.e., who was rated in the learning task, we tested *Group* (BPD/CON) and *Profile type* (BPD/CON) on other-ratings (see Supplementary Tables S16-18 for full model summaries and post-hoc contrasts). We found that the control group shows more favorable other-ratings for control profiles only (M_*BPD*_ = 5.48, SD_*BPD*_ = 1.44; M_*CON*_ = 5.73, SD_*CON*_ = 1.32; EMMs contrast: *b* = −0.24, *SE* = 0.08, *z* = −3.18, *p* < 0.01) but not for the BPD profiles (M_*BPD*_ = 5.41, SD_*BPD*_ = 1.40; M_*CON*_ = 5.40, SD_*CON*_ = 1.37; EMMs contrast: *b* = 0.01, *SE* = 0.08, *z* = 0.17, *p* = 0.863; see Fig. 3).

#### Prediction errors

**Fig. 4:**
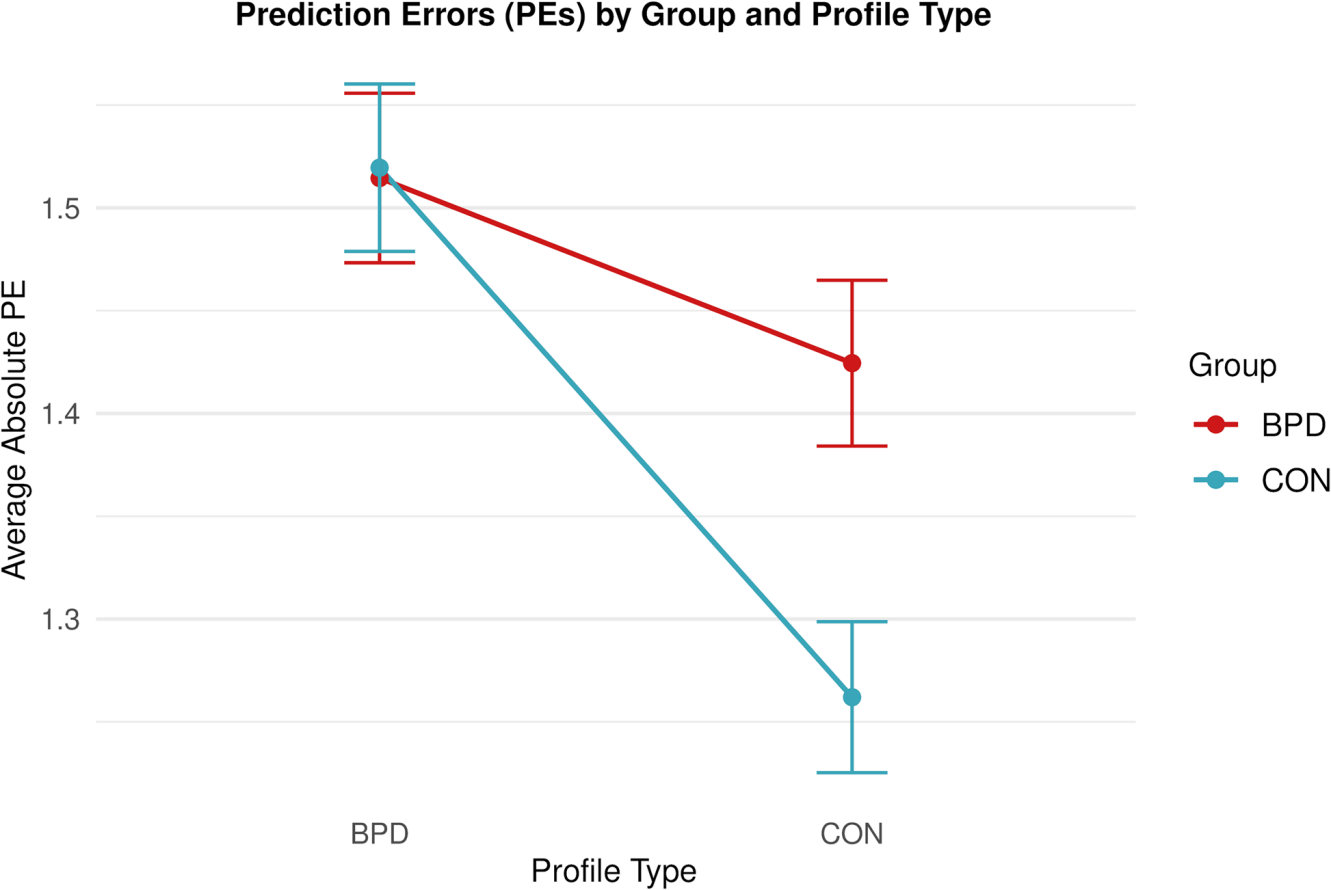
Average absolute PEs (across all trials) on BPD profiles and control profiles in the BPD group (red) and the control group (blue). The graph indicates that both groups show lower prediction errors in the control profiles, with the effect being more pronounced in the control group (significant Group and Profile Type interaction in an LME model and significant post-hoc contrasts for the effect of Group on the control profiles, but not for the BPD profiles). Lines show observed means, error bars indicate 95% confidence intervals based on a normal distribution

In line with our hypothesis [H3.1], we found that participants in the control group were more accurate in estimating the personality traits for control profiles than participants in the BPD group (Supplementary Tables S19-21 for full model summaries and post-hoc contrasts). Pairwise comparisons revealed that participants in the control group (*M*_*CON*_ = 1.26, *SD*_*CON*_ = 1.11) exhibited lower PEs for control profiles than participants in the BPD group (*M*_*BPD*_ = 1.42, *SD*_*BPD*_ = 1.21; EMMs contrast: *b* = 0.16, *SE* = 0.05, *z* = 3.00, *p* < 0.01). We additionally tested whether groups differed in estimating personality traits of BPD profiles and found no differences in PEs (M_*BPD*_ = 1.51, *SD*_*BPD*_ = 1.24; *M*_*CON*_ = 1.52, *SD*_*CON*_ = 1.25; EMMs contrast: *b* = −0.003, *SE* = 0.05, *z* = −0.06, *p* = 0.956). These exploratory findings suggest that group differences in PEs are specific to the control profiles, where the BPD group exhibits greater PEs than the control group (see Fig. 4).

Our hypothesis [H3.2] that individuals with BPD would be more accurate in estimating personality traits for BPD profiles than for control profiles was not supported by the data (see Supplementary Tables S19-20 and S22 for full model summaries and post-hoc contrasts). Pairwise comparisons revealed that individuals with BPD were more accurate in estimating personality traits for the control profiles (*M* = 1.42, *SD* = 1.21) than for BPD profiles (M = 1.51, *SD* = 1.24; *b =* 0.09, *SE* = 0.03, *z* = 3.32, *p* = 0.001). We found the same effect in the control group (CON profiles: M = 1.26, SD = 1.11; BPD profiles: M = 1.52, SD = 1.25; *b* = 0.26; *SE* = 0.03, *z* = 9.47, *p* < 0.001). Our findings suggest that both groups show lower prediction errors when learning about control profiles than when learning about BPD profiles, with the effect being more pronounced in the control group (see Fig. 4).

We tested whether PEs decreased over time, as an indication for learning, and found a small but significant negative effect of *Trial* (*b* = − 0.003, 95% *CI* [−0.005, −0.001], SE = 0.001, t_(13880)_ = − 3.10, *p* < 0.01) across participants and profiles (see Supplementary Tables S23-24 for full model summaries).

**Fig. 5:**
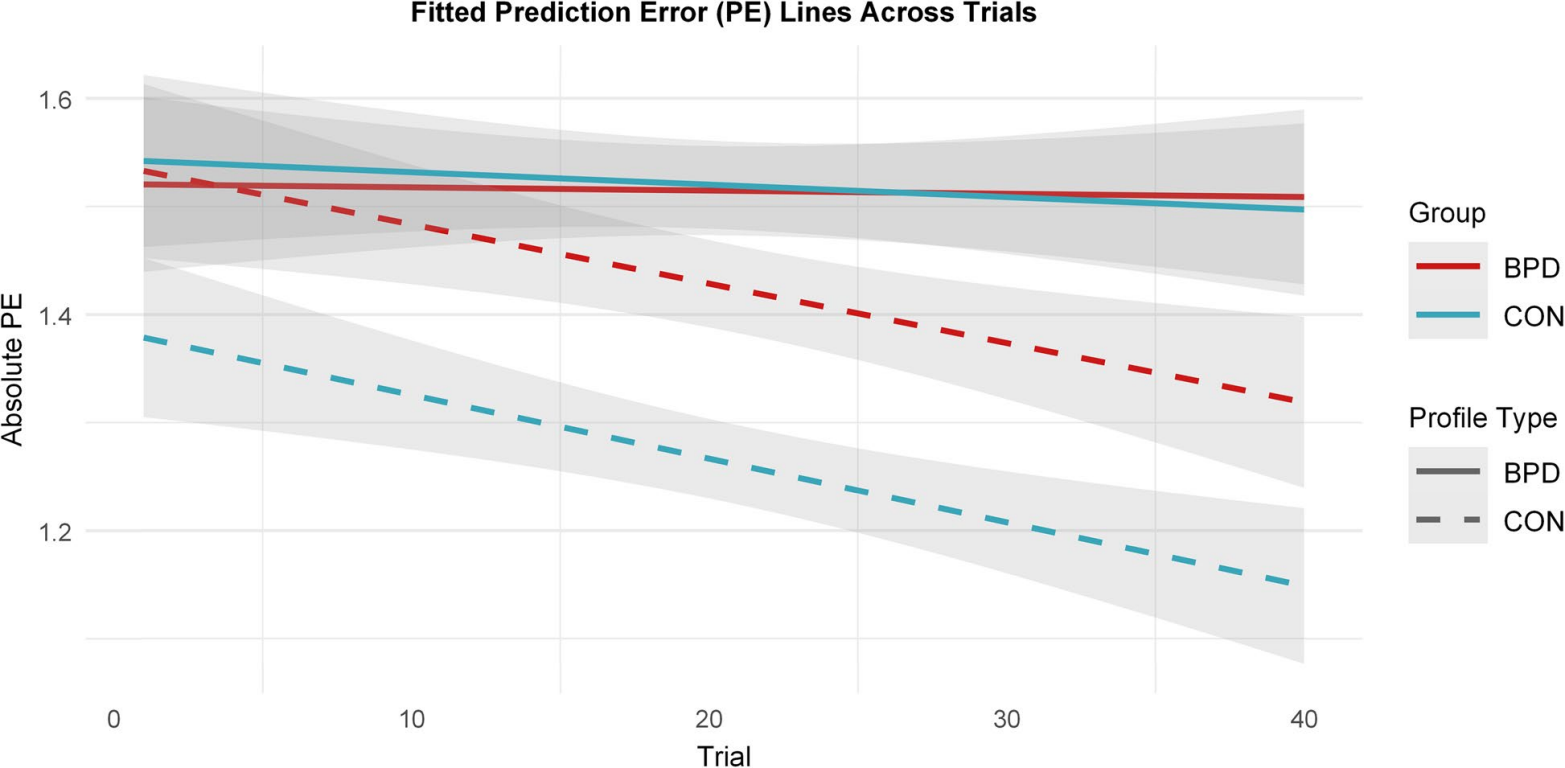
Fitted linear trends of PE reductions across trials for both groups (BPD in red vs. CON in blue) and profile types (BPD in solid lines vs. CON in dashed lines). The graph indicates that both groups exhibit reductions in PEs across trials when learning about control profiles, but not when learning about BPD profiles. In addition, the BPD group starts with higher PEs than the control group when learning about control profiles. Lines represent linear model fits of PEs across trials for each condition, with 95% confidence intervals (CIs) shaded around the lines. Note that individual data points are not shown; the lines represent model-predicted trends. Post-hoc tests of the LME model indicated that the slopes for control profiles were significantly different from zero for both groups, whereas the slopes for the BPD profiles were not. Post-hoc contrasts further revealed group differences for the control profiles, but not for the BPD profiles

In addition, we explored whether groups (BPD/CON) differed in PE reductions over time depending on the learning profile type (BPD/CON). We found that the PEs in both groups decreased significantly for the control profiles but not for the BPD profiles, as indicated by the 95% asymptotic confidence intervals that did not include zero (see Fig. 5). There were no group differences in PE reductions across or within learning profiles. The LME model summary and pairwise post-hoc tests for the slopes can be found in Supplementary Tables S25-S29.

### Computational models

**Fig. 6:**
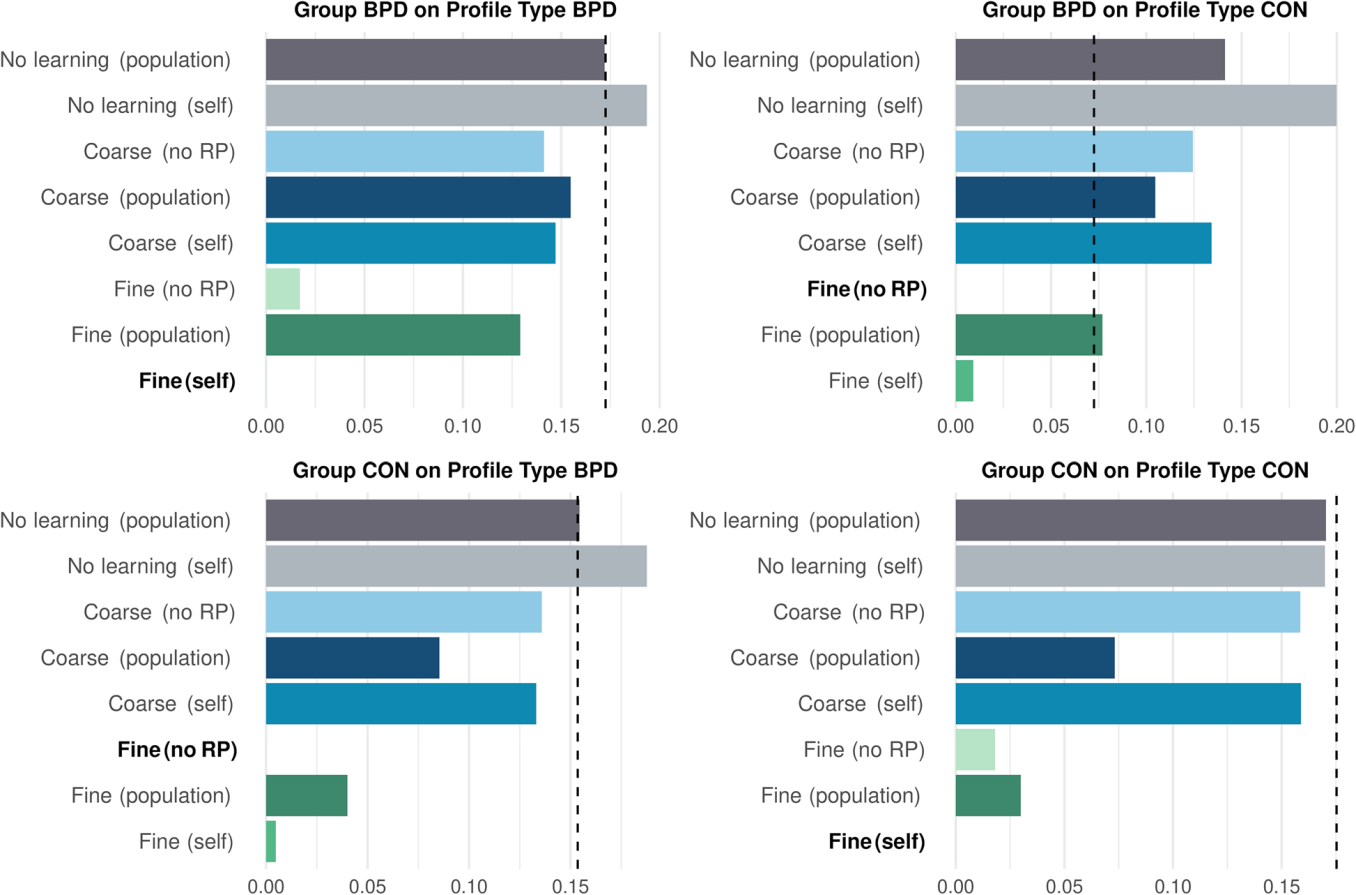
Barplots showing the Mean Squared Error (MSE) out-of-sample predictive performance differences of models compared to the best performing model across conditions (covering every pairing of profile and participant type). Performance differences were estimated by fitting eight models with different assumptions about the learning process to experimental data. The performance of a naive mean-prediction is given as a baseline (dashed line). No learning models are depicted in shades of gray, coarse-grained learning models in shades of blue, and fine-grained learning models in shades of green. Lower MSE values indicate better fit. The plots show that the fine granularity models outperform all other models in all conditions (see main text for complementary statistical analyses)

We tested learning in a more nuanced way by adapting and improving our previously established computational modeling framework [85]. We compared our previously introduced eight computational models in terms of their out-of-sample predictive accuracy as measured by their Mean Squared Error. The results of the model comparison are shown in Fig. 6. In short, our preregistered hypothesis [H4] of different winning models, depending on the two groups and the two types of learning profiles, could not be supported. The fine granularity model with self reference points and the one without reference points outperformed all other models (all ΔMSE >2SE) but did not credibly differentiate themselves from each other (all ΔMSE < 2SE). We therefore specifically used the “Fine (No RP)” model to address [H5], i.e., to test for differences in learning rates between the two groups. No credible differences in the learning rates emerged. The average V0 exclusively differed between the control-on-control vs. the BPD-on-control condition (Δ = 0.42, 95% SPI = [0.19, 0.65]. Supplementary Table S30 presents all parameter comparisons.

Given limitations in the design, we refrain from reporting the preregistered hypothesis [H6] about differences between learning rates for positive and negative PEs. We did not adequately consider these design limitations when writing the preregistration.

## Discussion

In this study, we examined whether women with BPD show negativity biases in trait assessments for self- and others and how these biases would affect their learning about other people’s personalities. As hypothesized, we found that the BPD group rated themselves more negatively than the control group. When rating BPD profiles, the groups did not differ in other-ratings, but when rating control profiles, control participants rated others more positively on personality traits compared to the BPD group. Control participants were also more accurate in estimating the personality traits for control profiles than participants in the BPD group. We did not find this “in-group effect” for BPD profiles. Both groups were similarly inaccurate when estimating personality traits for the BPD profiles. Relatedly, the PEs in both groups showed a small but significant decrease across time for the control profiles but not for the BPD profiles (although the difference in PE decrease did not reach significance). These findings suggest that BPD profiles are harder to learn about than control profiles. Comparing different computational models revealed that the fine granularity models with either self-reference points or no reference points exhibited the best fit in both groups regardless of profile. While these two models outperformed all other models, they could not be distinguished from each other.

### BPD group shows negatively biased self-ratings

Consistent with our hypothesis, individuals in the BPD group rated themselves more negatively across 40 personality traits compared to controls—reporting lower endorsement of positive traits and higher endorsement of negative traits. 

These findings are in line with previous studies showing that individuals with BPD describe themselves with more negative and less positive trait words [14, 34, 118, 119], and are consistent with a broader literature indicating a negative self-concept and heightened self-related negative emotions in BPD [35]. For instance, Winter et al. [120] found that individuals with BPD judged positive and neutral words less positively than healthy controls if the words referred to themselves, highlighting the role of self-referential processing. Moreover, numerous studies have demonstrated that BPD is associated with lower self-esteem [121–125], heightened levels of self-criticism [13], trait-shame proneness [126], and self-disgust [127–130].

These findings may reflect differences in self-motives: while healthy individuals are typically motivated to maintain a stable and positive self-concept, showing a greater propensity to internalize favorable feedback [29–32], individuals with BPD show a reduced or absent positivity bias and heightened sensitivity to unfavorable feedback [14, 33–35, 131]. Such heightened sensitivity to negative self-relevant information may reinforce negative self-perceptions, potentially contributing to emotional dysregulation [132] and interpersonal dysfunction [133], while hindering the formation of positive relationships [17].

To validate the observed differences in self-ratings on personality traits, we further assessed whether BPD participants rated themselves more negatively on two established personality measures: the NEO-FFI [36, 89] and the PID-5-BF [90–92]. On the NEO-FFI, individuals with BPD scored significantly higher than controls on Neuroticism—a dimension associated with emotional instability, anxiety, and self-consciousness. Consistent with prior findings, the BPD group also scored significantly lower on Extraversion, Agreeableness, and Conscientiousness—personality factors that are typically associated with socially desirable traits such as sociability, cooperativeness, and reliability. These results align with previous research showing that individuals with BPD tend to exhibit higher Neuroticism and lower Agreeableness and Conscientiousness compared to healthy controls [38, 39, 134, 135]. Lower Extraversion has also been reported, albeit with somewhat less consistency across studies [136, 137]. Openness, meanwhile, appears largely unrelated to BPD [38, 39].

When examining maladaptive personality traits using the PID-5-BF, individuals with BPD scored significantly higher than controls across all five pathological domains: Negative Affect, Detachment, Antagonism, Disinhibition, and Psychoticism. These results align with prior findings demonstrating that BPD is associated with elevated levels of maladaptive traits relative to both healthy and clinical comparison groups [41, 138]. Notably, PID-5 profiles in BPD are characterized by prominent features of negative affectivity and impulsivity, which are core aspects of the disorder’s clinical presentation and differ in other clinical groups [41, 138].

Taken together, our findings provide robust support for the hypothesis that individuals with BPD exhibit more negative self-views across a range of evaluative contexts. These pervasive negative self-evaluations may play a key role not only in the emotional but also in the interpersonal difficulties characteristic of BPD.

### Negative biases of individuals with BPD generalize to others

In contrast to our hypothesis, the groups did not differ significantly in their overall evaluations of others in the learning task. However, when dissociating profile types, we found that control participants evaluated control profiles more favorably than participants with BPD. In contrast, the BPD group showed comparable ratings for both BPD and control profiles. Importantly, while participants were unaware of whether the profiles were from patients with BPD or controls during the learning task, they did receive trial-by-trial feedback on the profiles in question. Our results suggest that the more negative evaluations of BPD profiles by BPD and control participants were influenced by the trait characteristics inherent to the profiles rather than by explicit knowledge or stereotypes about the BPD group. The group differences in how participants rated control profiles specifically showed that BPD participants maintained negative views of control profiles, despite receiving feedback that the other people in question rated themselves more favorably.

Our results are consistent with previous research indicating that individuals with BPD tend to view others as more negative and less trustworthy [14, 43, 51–55]. Notably, one study reported no differences in how individuals with BPD evaluated the valence of other-referential stimuli, suggesting that a negative bias for others may depend on whether the information is self-relevant or socially meaningful [120]. We found that both control and BPD participants rated BPD profiles similarly, indicating that the negative views align with the trait information they received on BPD profiles (which are inherently more negative). Previous research also showed that even in the absence of explicit trait feedback, participants evaluate individuals with BPD more negatively based on photographs and videos [27, 42–44]. We show that these negative evaluations are reinforced similarly through trait feedback, even in the absence of visual cues or diagnostic labels. This suggests that the trait pattern of people with BPD may lead to more negative impressions by others—even when others are unaware of the individual’s mental health status.

### BPD profiles are harder to learn than control profiles across groups

Both groups were more accurate in assessing control profiles than BPD profiles, and this effect was more pronounced in the control group. In line with our hypothesis, control participants were more accurate (i.e., showed slightly lower PEs) when evaluating control profiles than participants in the BPD group. However, contrary to our expectations, participants in the BPD group were not more accurate when evaluating BPD profiles compared to control profiles.

Participants in both groups became slightly more accurate in their personality trait assessment across trials, as indicated by a small, general decrease in PEs. Further analysis revealed that this effect was dependent on the profile type rather than the participant group. Specifically, both groups improved in accuracy when evaluating control profiles, but not when evaluating BPD profiles. Interestingly, the improvement (i.e., PE slopes) was similar across groups, which may suggest that learning about others based on feedback is similar in the BPD and control group. The BPD group started with lower initial accuracy (i.e., higher PEs), which may account for their overall reduced task performance. Surprisingly, BPD participants did not improve in accuracy when evaluating BPD profiles over time. This suggests that learning to assess personality traits in individuals with BPD is challenging not only for controls but also for those with BPD themselves. The absence of a group difference in improvement is noteworthy. Despite beginning with lower initial accuracy, individuals with BPD adapted their judgments over time to a similar extent as controls. Highlighting such intact capacities is important, as it underscores that social-cognitive processes in BPD are not uniformly impaired. Preserved feedback-based updating may provide a foundation for adaptive learning under conditions that are less complex and more realistic than the present task.

Although the overall effects were small, the observed decrease in PEs is consistent with a feedback-based learning process. Interpretation should nevertheless be cautious, given the modest size of the effects. As we discuss further in the limitations section, the small magnitude of these effects may partly reflect the complexity and limited realism of the current task design. Previous studies have shown that similar paradigms can capture social learning effects more robustly [80–83, 85], but given the demands of our paradigm, the current findings may be more cautiously interpreted as an improvement in accuracy that hints at an underlying learning process, rather than a strong confirmation of learning per se. Future refinements of the task design should help capture this process more clearly and with greater robustness.

### Both groups rely on a fine granularity learning model to improve predictions of others over time

Learning in both groups was best captured by models that integrate reinforcement learning with fine-grained social knowledge structures. Notably, models incorporating fine granularity consistently outperformed no learning models (which assume no belief updating over time) or coarse granularity models (which assume a generalized learning across broad personality dimensions), regardless of group or profile type.

Contrary to our initial hypothesis—that participants in the control group would rely on fine-grained similarity and participants in the BPD group on coarse-grained similarity—the results suggest that both groups used detailed knowledge of personality trait similarities when learning about others.

Using extended models, we tried to estimate participants’ initial expectations for each trait (V0 estimates), capturing prior beliefs about average trait expressions. Notably, average V0 values differed only credibly between groups when evaluating control profiles in the learning task.

While our modelling framework offers a nuanced way to assess social learning, the model comparisons did not suggest group differences in the learning process itself. Rather, individuals with BPD may be influenced more by their negative baseline expectations about themselves and others than by a dysfunctional learning process. Notably, this framework can be extended to other types of learning processes [16].

### Limitations and outlook

As with any experimental design, this study has several limitations that should be acknowledged.

First, the task employed in this study may have been overly difficult for participants. In future research, we will reduce complexity by presenting fewer profiles, allowing more time for responses, and selecting profiles that are more easily distinguishable. With these adjustments, we hope to ensure that the task better captures participants’ evaluation and learning processes.

Second, the current design lacked realism and direct social interactions, which limits its ecological validity. To address this, future studies should enhance the realism of the stimuli—for example, by including photographs and richer background information for each profile. In addition, incorporating more naturalistic approaches, such as live social interactions, could better approximate the dynamics of real-world personality learning. While our study emphasizes the importance of using a controlled baseline paradigm without confounding social cues, we also highlight the relevance of extending this work by incorporating more realistic stimuli to better capture the process of learning about other people’s personalities.

Third, as noted in the discussion, these design limitations may help explain why the learning effects observed in this study were small and why the exploratory power was limited. The overall weak learning effect might have limited our ability to detect significant group differences in the learning mechanisms. At the same time, even subtle effects can be informative in computational modeling, as they enable the estimation of underlying parameters and provide a basis for testing mechanistic hypotheses. An additional strength of this work is the introduction of a novel paradigm that integrates reinforcement learning with pre-existing knowledge in a clinical population. Our framework can be readily adapted to other populations and learning contexts. With further refinements to the task design, this paradigm may serve as a valuable starting point for systematically testing social learning processes in clinical populations.

Fourth, the sample consisted exclusively of female participants learning about female profiles, which limits the generalizability of the findings. Additionally, our sample size may limit statistical power and the detection of smaller effects. Nonetheless, a key strength of this study is the use of a well-characterized sample of women with BPD, carefully matched with controls on age and intelligence. This provides a foundation for future work using dimensional diagnostic approaches.

## Conclusion

Our study provides compelling evidence that individuals with BPD exhibit pervasive negative biases not only in their self-evaluations, but also in their perceptions of others’ personalities. Despite these biases, both groups relied on fine-grained social learning models, suggesting that the biased evaluations in individuals with BPD are likely driven by more negative baseline expectations rather than impaired learning mechanisms, although this conclusion warrants further investigation. The learning effects observed were modest and should be interpreted with caution, but the absence of a deficit in feedback-based updating is noteworthy. Individuals with BPD were able to adapt their judgments over time to a similar extent as controls, underscoring that their capacity for feedback-based social learning remains intact. Highlighting this preserved ability provides an important counterbalance to deficit-focused narratives. At the same time, profiles reflecting BPD traits remained consistently harder to assess accurately across groups, which may contribute to a self-reinforcing cycle: individuals with BPD hold more negative expectations about themselves and others, and are also more likely to be viewed negatively by others. This reciprocal negativity may exacerbate interpersonal difficulties. Importantly, the intact learning capacity observed suggests a potential therapeutic resource: interventions that target and recalibrate initial social expectations could help mitigate these biases and enhance social outcomes for individuals with BPD.

## Supplementary Information

Supplementary Material 1.

## Data Availability

The data and code for this study are publicly available on Github (https://github.com/dnhi-lab/PersonalityLearningBPD_2025).

## References

[1] Kandler C, Bleidorn W. Personality Differences and Development: Genetic and Environmental Contributions. Int Encycl Soc Behav Sci. Elsevier; 2015. p. 884–90. 10.1016/B978-0-08-097086-8.25011-3.

[2] Borkenau P, Liebler A. Trait inferences: Sources of validity at zero acquaintance. J Pers Soc Psychol. 1992;62:645–57. 10.1037/0022-3514.62.4.645.

[3] Bott A, Brockmann L, Denneberg I, Henken E, Kuper N, Kruse F, et al. Spontaneous Trait Inferences From Behavior: A Systematic Meta-Analysis. Pers Soc Psychol Bull. 2024;50:78–102. 10.1177/01461672221100336. 10.1177/01461672221100336 PMC10676050 35751144

[4] Carlston DE, Skowronski JJ. Savings in the relearning of trait information as evidence for spontaneous inference generation. J Pers Soc Psychol 1994;66:840–56. 10.1037/0022-3514.66.5.840.

[5] Funder DC. Accurate Personality Judgment. Curr Dir Psychol Sci. 2012;21:177–82. 10.1177/0963721412445309.

[6] Jaksic C, Schlegel K. Accuracy in Judging Others’ Personalities: The Role of Emotion Recognition, Emotion Understanding, and Trait Emotional Intelligence. J Intell. 2020;8:34. 10.3390/jintelligence8030034. 10.3390/jintelligence8030034 PMC7555973 32961916

[7] Little AC, Perrett DI. Using composite images to assess accuracy in personality attribution to faces. Br J Psychol. 2007;98:111–26. 10.1348/000712606X109648. 10.1348/000712606x109648 17319053

[8] Naumann LP, Vazire S, Rentfrow PJ, Gosling SD. Personality Judgments Based on Physical Appearance. Pers Soc Psychol Bull. 2009;35:1661–71. 10.1177/0146167209346309. 10.1177/0146167209346309 19762717

[9] Albright L, Kenny DA, Malloy TE. Consensus in personality judgments at zero acquaintance. J Pers Soc Psychol. 1988;55:387–95. 10.1037/0022-3514.55.3.387. 10.1037//0022-3514.55.3.387 3171912

[10] Cho JC, Knowles ED. I’m like you and you’re like me: Social projection and self-stereotyping both help explain self–other correspondence. J Pers Soc Psychol. 2013;104:444–56. 10.1037/a0031017. 10.1037/a0031017 23276270

[11] Thielmann I, Spadaro G, Balliet D. Personality and prosocial behavior: A theoretical framework and meta-analysis. Psychol Bull. 2020;146:30–90. 10.1037/bul0000217. 10.1037/bul0000217 31841013

[12] Wood D, Harms P, Vazire S. Perceiver effects as projective tests: What your perceptions of others say about you. J Pers Soc Psychol. 2010;99:174–90. 10.1037/a0019390. 10.1037/a0019390 20565194

[13] Kopala-Sibley DC, Zuroff DC, Russell JJ, Moskowitz DS, Paris J. Understanding heterogeneity in borderline personality disorder: Differences in affective reactivity explained by the traits of dependency and self-criticism. J Abnorm Psychol. 2012;121:680–91. 10.1037/a0028513. 10.1037/a0028513 22686873

[14] Korn CW, La Rosée L, Heekeren HR, Roepke S. Social feedback processing in borderline personality disorder. Psychol Med. 2016;46:575–87. 10.1017/S003329171500207X. 10.1017/S003329171500207X 26467724

[15] Niedtfeld I, Renkewitz F, Mädebach A, Hillmann K, Kleindienst N, Schmahl C, et al. Enhanced memory for negative social information in borderline personality disorder. J Abnorm Psychol. 2020;129:480–91. 10.1037/abn0000540. 10.1037/abn0000540 32437207

[16] Rosenblau G, Frolichs K, Korn CW. A neuro-computational social learning framework to facilitate transdiagnostic classification and treatment across psychiatric disorders. Neurosci Biobehav Rev. 2023;149:105181. 10.1016/j.neubiorev.2023.105181. 10.1016/j.neubiorev.2023.105181 PMC10236440 37062494

[17] Van Schie CC, Whiting L, Grenyer BFS. How negative self-views may interfere with building positive relationships: An experimental analogue of identity dysfunction in borderline personality disorder. PLOS ONE. 2024;19:e0301196. 10.1371/journal.pone.0301196. 10.1371/journal.pone.0301196 PMC10977689 38547086

[18] Werner AM, Tibubos AN, Rohrmann S, Reiss N. The clinical trait self-criticism and its relation to psychopathology: A systematic review – Update. J Affect Disord. 2019;246:530–47. 10.1016/j.jad.2018.12.069. 10.1016/j.jad.2018.12.069 30599378

[19] American Psychiatric Association. Diagnostic and Statistical Manual of Mental Disorders. Fifth Edition. American Psychiatric Association; 2013. 10.1176/appi.books.9780890425596.

[20] Gunderson JG, Herpertz SC, Skodol AE, Torgersen S, Zanarini MC. Borderline personality disorder. Nat Rev Dis Primer. 2018;4:18029. 10.1038/nrdp.2018.29. 10.1038/nrdp.2018.29 29795363

[21] Bouchard S, Sabourin S. Borderline personality disorder and couple dysfunctions. Curr Psychiatry Rep. 2009;11:55–62. 10.1007/s11920-009-0009-x. 10.1007/s11920-009-0009-x 19187710

[22] Lazarus SA, Cheavens JS, Festa F, Zachary Rosenthal M. Interpersonal functioning in borderline personality disorder: A systematic review of behavioral and laboratory-based assessments. Clin Psychol Rev. 2014;34:193–205. 10.1016/j.cpr.2014.01.007 24534643

[23] Liebke L, Bungert M, Thome J, Hauschild S, Gescher DM, Schmahl C, et al. Loneliness, social networks, and social functioning in borderline personality disorder. Personal Disord Theory Res Treat. 2017;8:349–56. 10.1037/per0000208. 10.1037/per0000208 27505189

[24] Stepp SD, Pilkonis PA, Yaggi KE, Morse JQ, Feske U. Interpersonal and Emotional Experiences of Social Interactions in Borderline Personality Disorder. J Nerv Ment Dis. 2009;197:484–91. 10.1097/NMD.0b013e3181aad2e7. 10.1097/NMD.0b013e3181aad2e7 PMC2757039 19597355

[25] Amestoy ME, Best MW, Ruocco AC, Uliaszek AA. Borderline personality disorder stigma: Examining the effects of diagnostic disclosure, behavior, and gender as sources of stigma in the general population. Personal Disord Theory Res Treat. 2024;15:254–63. 10.1037/per0000672. 10.1037/per0000672 38780568

[26] Aviram RB, Brodsky BS, Stanley B. Borderline Personality Disorder, Stigma, and Treatment Implications. Harv Rev Psychiatry. 2006;14:249–56. 10.1080/10673220600975121. 10.1080/10673220600975121 16990170

[27] Hepp J, Störkel LM, Kieslich PJ, Schmahl C, Niedtfeld I. Negative evaluation of individuals with borderline personality disorder at zero acquaintance. Behav Res Ther. 2018;111:84–91. 10.1016/j.brat.2018.09.009. 10.1016/j.brat.2018.09.009 30342222

[28] Herpertz SC, Schneider I, Renneberg B, Schneider A. Patients with personality disorders in everyday clinical practice—implications of the ICD-11. Dtsch Ärztebl Int. 2022. 10.3238/arztebl.m2022.0001. 10.3238/arztebl.m2022.0001 PMC9002298 34809749

[29] Elder JJ, Davis TH, Hughes BL. A Fluid Self-Concept: How the Brain Maintains Coherence and Positivity across an Interconnected Self-Concept While Incorporating Social Feedback. J Neurosci. 2023;43:4110–28. 10.1523/JNEUROSCI.1951-22.2023. 10.1523/JNEUROSCI.1951-22.2023 PMC10255005 37156606

[30] Hughes BL, Beer JS. Protecting the Self: The Effect of Social-evaluative Threat on Neural Representations of Self. J Cogn Neurosci. 2013;25:613–22. 10.1162/jocn_a_00343. 10.1162/jocn_a_00343 23249346

[31] Korn CW, Prehn K, Park SQ, Walter H, Heekeren HR. Positively Biased Processing of Self-Relevant Social Feedback. J Neurosci. 2012;32:16832–44. 10.1523/JNEUROSCI.3016-12.2012. 10.1523/JNEUROSCI.3016-12.2012 PMC6621762 23175836

[32] Leary MR. Motivational and Emotional Aspects of the Self. Annu Rev Psychol. 2007;58:317–44. 10.1146/annurev.psych.58.110405.085658. 10.1146/annurev.psych.58.110405.085658 16953794

[33] Van Schie CC, Chiu C-D, Rombouts SARB, Heiser WJ, Elzinga BM. Stuck in a negative me: fMRI study on the role of disturbed self-views in social feedback processing in borderline personality disorder. Psychol Med. 2020;50:625–35. 10.1017/S0033291719000448. 10.1017/S0033291719000448 PMC7093320 30867073

[34] Vater A, Schröder-Abé M, Weißgerber S, Roepke S, Schütz A. Self-concept structure and borderline personality disorder: Evidence for negative compartmentalization. J Behav Ther Exp Psychiatry. 2015;46:50–8. 10.1016/j.jbtep.2014.08.003. 10.1016/j.jbtep.2014.08.003 25222626

[35] Winter D, Bohus M, Lis S. Understanding Negative Self-Evaluations in Borderline Personality Disorder—a Review of Self-Related Cognitions, Emotions, and Motives. Curr Psychiatry Rep. 2017;19:17. 10.1007/s11920-017-0771-0. 10.1007/s11920-017-0771-0 28290062

[36] Costa PTJr, McCrae RR. Revised NEO Personality Inventory (NEO-PI-R) and NEO Five Factor Inventory Professional Manual. Psychological Assessment Resources; 1992.

[37] Goldberg LR. An alternative “description of personality”: The Big-Five factor structure. J Pers Soc Psychol. 1990;59:1216–29. 10.1037/0022-3514.59.6.1216. 10.1037//0022-3514.59.6.1216 2283588

[38] Samuel D, Widiger T. A meta-analytic review of the relationships between the five-factor model and DSM-IV-TR personality disorders: A facet level analysis☆. Clin Psychol Rev. 2008;28:1326–42. 10.1016/j.cpr.2008.07.002. 10.1016/j.cpr.2008.07.002 PMC2614445 18708274

[39] Saulsman LM, Page AC. The five-factor model and personality disorder empirical literature: A meta-analytic review. Clin Psychol Rev. 2004;23:1055–85. 10.1016/j.cpr.2002.09.001. 10.1016/j.cpr.2002.09.001 14729423

[40] D’Adda F, Sighinolfi G, Mitolo M, Scala M, Guidi L, Motta L, et al. Neurobiological correlates of personality dimensions in borderline personality disorder using graph analysis of functional connectivity. Sci Rep. 2025;15:12623. 10.1038/s41598-025-85989-x. 10.1038/s41598-025-85989-x PMC11993620 40221425

[41] Calvo N, Valero S, Sáez-Francàs N, Gutiérrez F, Casas M, Ferrer M. Borderline Personality Disorder and Personality Inventory for DSM-5 (PID-5): Dimensional personality assessment with DSM-5. Compr Psychiatry. 2016;70:105–11. 10.1016/j.comppsych.2016.07.002. 10.1016/j.comppsych.2016.07.002 27624429

[42] Daros AR, Ruocco AC, Rule NO. Identifying Mental Disorder from the Faces of Women with Borderline Personality Disorder. J Nonverbal Behav. 2016;40:255–81. 10.1007/s10919-016-0237-9.

[43] Hepp J, Kieslich PJ, Schmitz M, Schmahl C, Niedtfeld I. Negativity on two sides: Individuals with borderline personality disorder form negative first impressions of others and are perceived negatively by them. Personal Disord Theory Res Treat. 2021;12:514–25. 10.1037/per0000412. 10.1037/per0000412 32881574

[44] Oltmanns TF, Friedman JNW, Fiedler ER, Turkheimer E. Perceptions of people with personality disorders based on thin slices of behavior. J Res Personal. 2004;38:216–29. 10.1016/S0092-6566(03)00066-7.

[45] Bertsch K, Krauch M, Stopfer K, Haeussler K, Herpertz SC, Gamer M. Interpersonal Threat Sensitivity in Borderline Personality Disorder: An Eye-Tracking Study. J Personal Disord. 2017;31:647–70. 10.1521/pedi_2017_31_273. 10.1521/pedi_2017_31_273 28072041

[46] Bertsch K, Gamer M, Schmidt B, Schmidinger I, Walther S, Kästel T, et al. Oxytocin and Reduction of Social Threat Hypersensitivity in Women With Borderline Personality Disorder. Am J Psychiatry. 2013;170:1169–77. 10.1176/appi.ajp.2013.13020263. 10.1176/appi.ajp.2013.13020263 23982273

[47] Domes G, Czieschnek D, Weidler F, Berger C, Fast K, Herpertz SC. Recognition of Facial Affect in Borderline Personality Disorder. J Personal Disord. 2008;22:135–47. 10.1521/pedi.2008.22.2.135. 10.1521/pedi.2008.22.2.135 18419234

[48] Fenske S, Lis S, Liebke L, Niedtfeld I, Kirsch P, Mier D. Emotion recognition in borderline personality disorder: effects of emotional information on negative bias. Borderline Personal Disord Emot Dysregulation. 2015;2:10. 10.1186/s40479-015-0031-z. 10.1186/s40479-015-0031-z PMC4579484 26401312

[49] Izurieta Hidalgo NA, Oelkers-Ax R, Nagy K, Mancke F, Bohus M, Herpertz SC, et al. Time course of facial emotion processing in women with borderline personality disorder: an ERP study. J Psychiatry Neurosci. 2016;41:16–26. 10.1503/jpn.140215. 10.1503/jpn.140215 PMC4688024 26269211

[50] Thome J, Liebke L, Bungert M, Schmahl C, Domes G, Bohus M, et al. Confidence in facial emotion recognition in borderline personality disorder. Personal Disord Theory Res Treat. 2016;7:159–68. 10.1037/per0000142. 10.1037/per0000142 26389624

[51] Barnow S, Stopsack M, Grabe HJ, Meinke C, Spitzer C, Kronmüller K, et al. Interpersonal evaluation bias in borderline personality disorder. Behav Res Ther. 2009;47:359–65. 10.1016/j.brat.2009.02.003. 10.1016/j.brat.2009.02.003 19278670

[52] Hepp J, Kieslich PJ, Wycoff AM, Bertsch K, Schmahl C, Niedtfeld I. Mouse-tracking reveals cognitive conflict during negative impression formation in women with BorderlinePersonality Disorder or Social Anxiety Disorder. PLOS ONE. 2021;16:e0247955. 10.1371/journal.pone.0247955. 10.1371/journal.pone.0247955 PMC7932102 33662030

[53] Scheunemann J, Jelinek L, Biedermann SV, Lipp M, Yassari AH, Kühn S, et al. Can you trust this source? Advice taking in borderline personality disorder. Eur Arch Psychiatry Clin Neurosci. 2023;273:875–85. 10.1007/s00406-022-01539-w. 10.1007/s00406-022-01539-w PMC10238350 36629942

[54] Lévay EE, Bajzát B, Unoka ZS. Expectation of Selfishness From Others in Borderline Personality Disorder. Front Psychol. 2021;12:702227. 10.3389/fpsyg.2021.702227. 10.3389/fpsyg.2021.702227 PMC8416988 34489805

[55] Masland SR, Schnell SE, Shah TV. Trust Beliefs, Biases, and Behaviors in Borderline Personality Disorder: Empirical Findings and Relevance to Epistemic Trust. Curr Behav Neurosci Rep. 2020;7:239–49. 10.1007/s40473-020-00220-7.

[56] Guest O, Martin AE. How Computational Modeling Can Force Theory Building in Psychological Science. Perspect Psychol Sci. 2021;16:789–802. 10.1177/1745691620970585. 10.1177/1745691620970585 33482070

[57] Van Rooij I, Baggio G. Theory Before the Test: How to Build High-Verisimilitude Explanatory Theories in Psychological Science. Perspect Psychol Sci. 2021;16:682–97. 10.1177/1745691620970604. 10.1177/1745691620970604 PMC8273840 33404356

[58] Lewandowsky S, Farrell S. Computational Modeling in Cognition: Principles and Practice. 2455 Teller Road, Thousand Oaks California 91320 United States: SAGE Publications, Inc.; 2011. 10.4135/9781483349428.

[59] Niv Y, Langdon A. Reinforcement learning with Marr. Curr Opin Behav Sci. 2016;11:67–73. 10.1016/j.cobeha.2016.04.005. 10.1016/j.cobeha.2016.04.005 PMC4939081 27408906

[60] Sutton RS, Barto A. Reinforcement learning: an introduction. Nachdruck. Cambridge, Massachusetts: The MIT Press; 2014.

[61] Montague P, Dayan P, Sejnowski T. A framework for mesencephalic dopamine systems based on predictive Hebbian learning. J Neurosci. 1996;16:1936–47. 10.1523/JNEUROSCI.16-05-01936.1996. 10.1523/JNEUROSCI.16-05-01936.1996 PMC6578666 8774460

[62] Schultz W, Apicella P, Ljungberg T. Responses of monkey dopamine neurons to reward and conditioned stimuli during successive steps of learning a delayed response task. J Neurosci. 1993;13:900–13. 10.1523/JNEUROSCI.13-03-00900.1993. 10.1523/JNEUROSCI.13-03-00900.1993 PMC6576600 8441015

[63] Pagnoni G, Zink CF, Montague PR, Berns GS. Activity in human ventral striatum locked to errors of reward prediction. Nat Neurosci, 2002;5:97–8. 10.1038/nn802. 10.1038/nn802 11802175

[64] Schönberg T, Daw ND, Joel D, O’Doherty JP. Reinforcement Learning Signals in the Human Striatum Distinguish Learners from Nonlearners during Reward-Based Decision Making. J Neurosci. 2007;27:12860–7. 10.1523/JNEUROSCI.2496-07.2007. 10.1523/JNEUROSCI.2496-07.2007 PMC6673291 18032658

[65] Niv Y. Reinforcement learning in the brain. J Math Psychol. 2009;53:139–54. 10.1016/j.jmp.2008.12.005.

[66] Charpentier CJ, O’Doherty JP. The application of computational models to social neuroscience: promises and pitfalls. Soc Neurosci. 2018;13:637–47. 10.1080/17470919.2018.1518834. 10.1080/17470919.2018.1518834 PMC6309617 30173633

[67] Chierchia G, Soukupová M, Kilford EJ, Griffin C, Leung J, Palminteri S, et al. Confirmatory reinforcement learning changes with age during adolescence. Dev Sci. 2023;26:e13330. 10.1111/desc.13330. 10.1111/desc.13330 PMC7615280 36194156

[68] Dayan P, Niv Y. Reinforcement learning: The Good, The Bad and The Ugly. Curr Opin Neurobiol. 2008;18:185–96. 10.1016/j.conb.2008.08.003. 10.1016/j.conb.2008.08.003 18708140

[69] Lockwood PL, Klein-Flügge MC. Computational modelling of social cognition and behaviour—a reinforcement learning primer. Soc Cogn Affect Neurosci. 2020:nsaa040. 10.1093/scan/nsaa040. 10.1093/scan/nsaa040 PMC8343561 32232358

[70] Rodriguez Buritica JM, Eppinger B, Heekeren HR, Crone EA, Van Duijvenvoorde ACK. Observational reinforcement learning in children and young adults. Npj Sci Learn. 2024;9:18. 10.1038/s41539-024-00227-9. 10.1038/s41539-024-00227-9 PMC10937639 38480747

[71] Zhang L, Lengersdorff L, Mikus N, Gläscher J, Lamm C. Using reinforcement learning models in social neuroscience: frameworks, pitfalls and suggestions of best practices. Soc Cogn Affect Neurosci. 2020;15:695–707. 10.1093/scan/nsaa089. 10.1093/scan/nsaa089 PMC7393303 32608484

[72] Czekalla N, Schröder A, Mayer AV, Stierand J, Stolz DS, Kube T, et al. Neurocomputational Mechanisms Underlying Maladaptive Self-Belief Formation in Depression. 2024. 10.1101/2024.05.09.593087.

[73] Liebenow B, Jones R, DiMarco E, Trattner JD, Humphries J, Sands LP, et al. Computational reinforcement learning, reward (and punishment), and dopamine in psychiatric disorders. Front Psychiatry. 2022;13:886297. 10.3389/fpsyt.2022.886297. 10.3389/fpsyt.2022.886297 PMC9630918 36339844

[74] Maia TV, Frank MJ. From reinforcement learning models to psychiatric and neurological disorders. Nat Neurosci. 2011;14:154–62. 10.1038/nn.2723. 10.1038/nn.2723 PMC4408000 21270784

[75] Ossola P, Garrett N, Biso L, Bishara A, Marchesi C. Anhedonia and sensitivity to punishment in schizophrenia, depression and opiate use disorder. J Affect Disord. 2023;330:319–28. 10.1016/j.jad.2023.02.120. 10.1016/j.jad.2023.02.120 36889442

[76] Pike AC, Robinson OJ. Reinforcement Learning in Patients With Mood and Anxiety Disorders vs Control Individuals: A Systematic Review and Meta-analysis. JAMA Psychiatry. 2022;79:313. 10.1001/jamapsychiatry.2022.0051. 10.1001/jamapsychiatry.2022.0051 PMC8892374 35234834

[77] Reiter AMF, Heinze H-J, Schlagenhauf F, Deserno L. Impaired Flexible Reward-Based Decision-Making in Binge Eating Disorder: Evidence from Computational Modeling and Functional Neuroimaging. Neuropsychopharmacology. 2017;42:628–37. 10.1038/npp.2016.95. 10.1038/npp.2016.95 PMC5240187 27301429

[78] Schuermann B, Kathmann N, Stiglmayr C, Renneberg B, Endrass T. Impaired decision making and feedback evaluation in borderline personality disorder. Psychol Med. 2011;41:1917–27. 10.1017/S003329171000262X. 10.1017/S003329171000262X 21262034

[79] Waite EE, Savalia T, Cohen AL, Haliczer LA, Huffman S, Dixon-Gordon KL. Borderline personality disorder and learning: The influences of emotional state and social versus nonsocial feedback. J Affect Disord. 2024;363:474–82. 10.1016/j.jad.2024.07.072. 10.1016/j.jad.2024.07.072 39032716

[80] Diaconescu AO, Mathys C, Weber LAE, Kasper L, Mauer J, Stephan KE. Hierarchical prediction errors in midbrain and septum during social learning. Soc Cogn Affect Neurosci. 2017;12:618–34. 10.1093/scan/nsw171. 10.1093/scan/nsw171 PMC5390746 28119508

[81] Zaki J, Kallman S, Wimmer GE, Ochsner K, Shohamy D. Social Cognition as Reinforcement Learning: Feedback Modulates Emotion Inference. J Cogn Neurosci. 2016;28:1270–82. 10.1162/jocn_a_00978. 10.1162/jocn_a_00978 27167401

[82] Fareri DS, Chang LJ, Delgado MR. Computational Substrates of Social Value in Interpersonal Collaboration. J Neurosci. 2015;35:8170–80. 10.1523/JNEUROSCI.4775-14.2015. 10.1523/JNEUROSCI.4775-14.2015 PMC4444540 26019333

[83] Rosenblau G, Korn CW, Dutton A, Lee D, Pelphrey KA. Neurocognitive Mechanisms of Social Inferences in Typical and Autistic Adolescents. Biol Psychiatry Cogn Neurosci Neuroimaging. 2021;6:782–91. 10.1016/j.bpsc.2020.07.002. 10.1016/j.bpsc.2020.07.002 32952091

[84] Hackel LM, Kalkstein DA, Mende-Siedlecki P. Simplifying social learning. Trends Cogn Sci. 2024;28:428–40. 10.1016/j.tics.2024.01.004. 10.1016/j.tics.2024.01.004 38331595

[85] Frolichs KMM, Rosenblau G, Korn CW. Incorporating social knowledge structures into computational models. Nat Commun. 2022;13:6205. 10.1038/s41467-022-33418-2. 10.1038/s41467-022-33418-2 PMC9584930 36266284

[86] Stolier RM, Hehman E, Freeman JB. Trait knowledge forms a common structure across social cognition. Nat Hum Behav. 2020;4:361–71. 10.1038/s41562-019-0800-6. 10.1038/s41562-019-0800-6 31932689

[87] Thornton MA, Tamir DI. The Organization of Social Knowledge Is Tuned for Prediction. In: Gilead M, Ochsner KN, editors. Neural Basis Ment., Cham: Springer International Publishing; 2021. p. 283–97. 10.1007/978-3-030-51890-5_14.

[88] Rosenblau G, Korn CW, Pelphrey KA. A Computational Account of Optimizing Social Predictions Reveals That Adolescents Are Conservative Learners in Social Contexts. J Neurosci. 2018;38:974–88. 10.1523/JNEUROSCI.1044-17.2017. 10.1523/JNEUROSCI.1044-17.2017 PMC5783970 29255008

[89] Borkenau P, Ostendorf F. NEO-Fünf-Faktoren-Inventar nach Costa und McCrae (NEO-FFI; 2. neu normierte und vollständig überarb. Aufl.). Göttingen: Hogrefe; 2007.

[90] American Psychiatric Association. The Personality Inventory for DSM–5—Brief Form (PID-5-BF)—Adult. Available from: https://www.psychiatry.org/File%20Library/Psychiatrists/Practice/DSM/APA_DSM5_The-Personality-Inventory-For-DSM-5-Brief-Form-Adult.pdf.

[91] Krueger RF, Derringer J, Markon KE, Watson D, Skodol AE. Initial construction of a maladaptive personality trait model and inventory for DSM-5. Psychol Med. 2012;42:1879–90. 10.1017/S0033291711002674. 10.1017/S0033291711002674 PMC3413381 22153017

[92] Zimmermann J, Altenstein D, Krieger T, Holtforth MG, Pretsch J, Alexopoulos J, et al. The Structure and Correlates of Self-Reported DSM-5 Maladaptive Personality Traits: Findings From Two German-Speaking Samples. J Personal Disord. 2014;28:518–40. 10.1521/pedi_2014_28_130. 10.1521/pedi_2014_28_130 24511899

[93] Doppelhofer LM, Löloff J, Neukel C, Herpertz SC, Korn CW. Cooperative decision-making in borderline personality disorder: insights from a preregistered study using a comprehensive economic task battery. Borderline Personal Disord Emot Dysregul. 2025;12(1):24. 10.1186/s40479-025-00295-2. 10.1186/s40479-025-00295-2 PMC12172245 40528240

[94] Leiner DJ. SoSci Survey (Version 3.1.06) 2019.

[95] Margraf J, Cwik JC. Mini-DIPS Open Access: Diagnostisches Kurzinterview bei psychischen Störungen. 2017. 10.13154/RUB.102.91.

[96] Loranger AW, Sartorius N, Andreoli A. The International Personality Disorder Examination: The World Health Organization/Alcohol, Drug Abuse, and Mental Health Administration International Pilot Study of Personality Disorders. Arch Gen Psychiatry. 1994;51:215. 10.1001/archpsyc.1994.03950030051005. 10.1001/archpsyc.1994.03950030051005 8122958

[97] Loranger AW, Sartorius N, Andreoli A, Berger P, Buchheim P, Channabasavanna SM, et al. Deutschsprachige Fassung der International personality disorder examination: IPDE. Genf WHO; 1998. 10.1001/archpsyc.1994.03950030051005 8122958

[98] Baudson TG, Preckel F. mini-q: Intelligenzscreening in drei Minuten. Diagnostica. 2016;62:182–97. 10.1026/0012-1924/a000150.

[99] Kuznetsova A, Brockhoff PB, Christensen RHB. lmerTest Package: Tests in Linear Mixed Effects Models. J Stat Softw. 2017;82. 10.18637/jss.v082.i13.

[100] Lüdecke D. sjPlot: Data Visualization for Statistics in Social Science. 2024.

[101] Lenth RV. emmeans: Estimated Marginal Means, aka Least-Squares Means. 2024. https://CRAN.R-project.org/package=emmeans

[102] Torchiano M. Effsize - a package for efficient effect size computation. 2016. 10.5281/ZENODO.1480624.

[103] Degenholtz HB, Bhatnagar M. Introduction to Hierarchical Modeling. J Palliat Med. 2009;12:631–8. 10.1089/jpm.2009.9595. 10.1089/jpm.2009.9595 19594348

[104] Gelman A. Multilevel (Hierarchical) Modeling: What It Can and Cannot Do. Technometrics. 2006;48:432–5. 10.1198/004017005000000661.

[105] Haines N, Kvam PD, Irving L, Smith CT, Beauchaine TP, Pitt MA et al. A tutorial on using generative models to advance psychological science: lessons from the reliability paradox. Psychol Methods. 2025. 10.1037/met0000674. 10.1037/met0000674 40232753

[106] Suppes P. Models of Data. Stud. Log. Found. Math., vol. 44, Elsevier; 1966, p. 252–61. 10.1016/S0049-237X(09)70592-0.

[107] White CN, Ratcliff R, Starns JJ. Diffusion models of the flanker task: Discrete versus gradual attentional selection. Cognit Psychol. 2011;63:210–38. 10.1016/j.cogpsych.2011.08.001. 10.1016/j.cogpsych.2011.08.001 PMC3195995 21964663

[108] Wiecki TV, Sofer I, Frank MJ. HDDM: Hierarchical Bayesian estimation of the Drift-Diffusion Model in Python. Front Neuroinformatics. 2013;7. 10.3389/fninf.2013.00014. 10.3389/fninf.2013.00014 PMC3731670 23935581

[109] Estes WK. The problem of inference from curves based on group data. Psychol Bull. 1956;53:134–40. 10.1037/h0045156. 10.1037/h0045156 13297917

[110] Farrell S, Lewandowsky S. Computational Modeling of Cognition and Behavior: 1st ed. Cambridge University Press; 2018. 10.1017/CBO9781316272503.

[111] Turner BM, Forstmann BU, Love BC, Palmeri TJ, Van Maanen L. Approaches to analysis in model-based cognitive neuroscience. J Math Psychol. 2017;76:65–79. 10.1016/j.jmp.2016.01.001. 10.1016/j.jmp.2016.01.001 PMC6863443 31745373

[112] Whelan R. Effective Analysis of Reaction Time Data. Psychol Rec. 2008;58:475–82. 10.1007/bf03395630.

[113] Carpenter B, Gelman A, Hoffman MD, Lee D, Goodrich B, Betancourt M, et al. Stan: A Probabilistic Programming Language. J Stat Softw. 2017;76. 10.18637/jss.v076.i01. 10.18637/jss.v076.i01 PMC9788645 36568334

[114] Gabry J, Češnovar R, Johnson A, Bronder S. cmdstanr: R Interface to “CmdStan.” 2025.

[115] Lee MD, Wagenmakers E-J. Bayesian cognitive modeling: a practical course. Cambridge, United Kingdom New York: Cambridge University Press; 2014. 10.1017/CBO9781139087759.

[116] Vehtari A, Gelman A, Gabry J. Practical Bayesian model evaluation using leave-one-out cross-validation and WAIC. 2015. 10.48550/ARXIV.1507.04544.

[117] Loken E, Gelman A. Measurement error and the replication crisis. Science. 2017;355:584–5. 10.1126/science.aal3618. 10.1126/science.aal3618 28183939

[118] Auerbach RP, Tarlow N, Bondy E, Stewart JG, Aguirre B, Kaplan C, et al. Electrocortical Reactivity During Self-referential Processing in Female Youth With Borderline Personality Disorder. Biol Psychiatry Cogn Neurosci Neuroimaging. 2016;1:335–44. 10.1016/j.bpsc.2016.04.004. 10.1016/j.bpsc.2016.04.004 PMC5472065 28626812

[119] Beeney JE, Hallquist MN, Ellison WD, Levy KN. Self–other disturbance in borderline personality disorder: Neural, self-report, and performance-based evidence. Personal Disord Theory Res Treat. 2016;7:28–39. 10.1037/per0000127. 10.1037/per0000127 PMC4659768 26011577

[120] Winter D, Herbert C, Koplin K, Schmahl C, Bohus M, Lis S. Negative Evaluation Bias for Positive Self-Referential Information in Borderline Personality Disorder. PLOS ONE. 2015;10:e0117083. 10.1371/journal.pone.0117083. 10.1371/journal.pone.0117083 PMC4303263 25612212

[121] Bungert M, Liebke L, Thome J, Haeussler K, Bohus M, Lis S. Rejection sensitivity and symptom severity in patients with borderline personality disorder: effects of childhood maltreatment and self-esteem. Borderline Personal Disord Emot Dysregulation. 2015;2:4. 10.1186/s40479-015-0025-x. 10.1186/s40479-015-0025-x PMC4579499 26401307

[122] Kanter JW, Parker CR, Kohlenberg RJ. Finding the self: A behavioral measure and its clinical implications. Psychother Theory Res Pract Train. 2001;38:198–211. 10.1037/0033-3204.38.2.198.

[123] Roepke S, Schröder‐Abé M, Schütz A, Jacob G, Dams A, Vater A, et al. Dialectic behavioural therapy has an impact on self‐concept clarity and facets of self‐esteem in women with borderline personality disorder. Clin Psychol Psychother. 2011;18:148–58. 10.1002/cpp.684. 10.1002/cpp.684 20187169

[124] Rüsch N, Lieb K, Göttler I, Hermann C, Schramm E, Richter H, et al. Shame and Implicit Self-Concept in Women With Borderline Personality Disorder. Am J Psychiatry. 2007;164:500–8. 10.1176/ajp.2007.164.3.500. 10.1176/ajp.2007.164.3.500 17329476

[125] Abela JRZ, Payne AVL, Moussaly N. Cognitive Vulnerability to Depression in Individuals With Borderline Personality Disorder. J Personal Disord. 2003;17:319–29. 10.1016/j.comppsych.2017.09.003 10.1521/pedi.17.4.319.23968 14521180

[126] Ritter K, Vater A, Rüsch N, Schröder-Abé M, Schütz A, Fydrich T, et al. Shame in patients with narcissistic personality disorder. Psychiatry Res. 2014;215:429–37. 10.1016/j.psychres.2013.11.019. 10.1016/j.psychres.2013.11.019 24321228

[127] Abdul-Hamid S, Denman C, Dudas RB. Self-Relevant Disgust and Self-Harm Urges in Patients with Borderline Personality Disorder and Depression: A Pilot Study with a Newly Designed Psychological Challenge. PLoS ONE. 2014;9:e99696. 10.1371/journal.pone.0099696. 10.1371/journal.pone.0099696 PMC4067282 24956153

[128] Clarke A, Simpson J, Varese F. A systematic review of the clinical utility of the concept of self‐disgust. Clin Psychol Psychother. 2019;26:110–34. 10.1002/cpp.2335. 10.1002/cpp.2335 30251455

[129] Dudas RB, Mole TB, Morris LS, Denman C, Hill E, Szalma B, et al. Amygdala and dlPFC abnormalities, with aberrant connectivity and habituation in response to emotional stimuli in females with BPD. J Affect Disord. 2017;208:460–6. 10.1016/j.jad.2016.10.043. 10.1016/j.jad.2016.10.043 27838143

[130] Schienle A, Leutgeb V, Wabnegger A. Symptom severity and disgust-related traits in borderline personality disorder: The role of amygdala subdivisions. Psychiatry Res Neuroimaging. 2015;232:203–7. 10.1016/j.pscychresns.2015.04.002. 10.1016/j.pscychresns.2015.04.002 25937342

[131] Klein MH, Wonderlich SA, Crosby R. Self-Concept Correlates of the Personality Disorders. J Personal Disord. 2001;15:150–6. 10.1521/pedi.15.2.150.19214. 10.1521/pedi.15.2.150.19214 11345850

[132] Neacsiu AD, Herr NR, Fang CM, Rodriguez MA, Rosenthal MZ. Identity Disturbance and Problems With Emotion Regulation Are Related Constructs Across Diagnoses. J Clin Psychol. 2015;71:346–61. 10.1002/jclp.22141. 10.1002/jclp.22141 25534425

[133] Gunderson JG, Lyons-Ruth K. BPD’s Interpersonal Hypersensitivity Phenotype: A Gene-Environment-Developmental Model. J Personal Disord. 2008;22:22–41. 10.1521/pedi.2008.22.1.22. 10.1521/pedi.2008.22.1.22 PMC2596628 18312121

[134] Dukalski B, Suslow T, Egloff B, Kersting A, Donges U-S. Implicit and explicit self-concept of neuroticism in borderline personality disorder. Nord J Psychiatry. 2019;73:159–68. 10.1080/08039488.2019.1582694. 10.1080/08039488.2019.1582694 30896322

[135] Wischniewski J, Brüne M. How Do People With Borderline Personality Disorder Respond to Norm Violations? Impact of Personality Factors on Economic Decision-Making. J Personal Disord. 2013;27:531–46. 10.1521/pedi_2012_26_036. 10.1521/pedi_2012_26_036 23130809

[136] Fowler CJ, Sharp C, Kalpakci A, Madan A, Clapp J, Allen JG, et al. A dimensional approach to assessing personality functioning: examining personality trait domains utilizing DSM-IV personality disorder criteria. Compr Psychiatry. 2015;56:75–84. 10.1016/j.comppsych.2014.09.001. 10.1016/j.comppsych.2014.09.001 25261890

[137] Zweig-Frank H, Paris J. The five-factor model of personality in borderline and nonborderline personality disorders. Can J Psychiatry. 1995;40(9):523–6. 8574987 10.1177/070674379504000904

[138] Fowler JC, Madan A, Allen JG, Patriquin M, Sharp C, Oldham JM, et al. Clinical utility of the DSM-5 alternative model for borderline personality disorder: differential diagnostic accuracy of the BFI, SCID-II-PQ, and PID-5. Compr Psychiatry. 2018;80:97–103. 29069625 10.1016/j.comppsych.2017.09.003

