# Supplementary material for: Estimating and learning personality traits of and from women with borderline personality disorder
Source: Borderline Personal Disord Emot Dysregul. 2026 Feb 9;13:5. doi: 10.1186/s40479-025-00320-4 (PMC12888613; doi:10.1186/s40479-025-00320-4)
Supplement: Supplementary file 1 — Supplementary Material 1. [file 40479_2025_320_MOESM1_ESM.docx]

**Supplementary material**

# **Supplementary methods**

## **Power analysis**

We conducted a power analysis using G*Power version 3.1.9.2 (1) to estimate the required sample size, based on a previous study (2) on group differences in updating undesirable feedback in individuals with BPD (N = 22) and controls (N = 42). The effect size reported in Korn et al. (2) was d = 0.65. Using an alpha level of .05 and a power of 1-β = .80, the analysis indicated a minimum sample size of N = 30 per group for a one-tailed two-sample t-test.

## **Participants**

### **Distribution of BPD criteria according to the IPDE**

In the BPD group, endorsement was highest for recurrent suicidal behavior/self-harm (90%), followed by impulsivity (80%), unstable and intense relationships (73.33%), affective instability (76.67%), chronic feelings of emptiness (73.33%). Moderate endorsement appeared for stress-related paranoid ideation or dissociation (60%), frantic efforts to avoid abandonment (56.67%), and identity disturbance (56.57%), while inappropriate/intense anger was least frequent (26.67%). In this sample the controls showed no endorsement (0%) for any of the nine criteria.

**Table S1** Comorbid diagnoses of the BPD group according to the mini-DIPS diagnostic interview

| Comorbid Diagnosis | *N* | **%** |
| --- | --- | --- |
| Panic disorder | 4 | 13.33 |
| Agoraphobia with panic | 1 | 3.33 |
| Specific phobia | 5 | 16.67 |
| Social anxiety disorder | 13 | 43.33 |
| Major depressive episode | 2 | 6.67 |
| Major depressive disorder, recurrent | 15 | 50 |
| Persistent depressive disorder | 3 | 10 |
| Obsessive-compulsive disorder | 1 | 3.33 |
| Body dysmorphic disorder | 1 | 3.33 |
| Posttraumatic stress disorder | 10 | 33.33 |
| Bulimia nervosa | 1 | 3.33 |
| Binge eating disorder | 2 | 6.67 |
| Illness anxiety disorder | 1 | 3.33 |
| Substance abuse, alcohol | 1 | 3.33 |
| Substance abuse, cannabis | 2 | 6.67 |
| No axis I diagnosis | 4 | 13.33 |

**Table S2** Psychotropic medication intake of the BPD group

| Medication | *N* | **%** |
| --- | --- | --- |
| Antidepressant, SNRI | 4 | 13.33 |
| Antidepressant, SSRI | 10 | 33.33 |
| Atypical antipsychotics | 3 | 10 |
| Mood stabilizer (Lamotrigine) | 2 | 6.67 |
| No medication | 14 | 46.67 |

## **Task instructions and additional information**

### ***Task instructions for the personality learning task (other-ratings)***

The following task instructions are translated from the German on-screen instructions:

In this task, you are supposed to evaluate six different people Imagine that you could meet these people in the future. First, you will see a short description of a person. After that, you should evaluate the person on various personality adjectives. After a fixation cross, you will see how the person has evaluated themselves. You can then compare your evaluation with the person’s self-evaluation.

Please give your rating on a scale from 1 to 8.

- 1 means: The trait does NOT apply at all.
- 8 means: The trait applies VERY STRONGLY.

At the beginning, of course, you know almost nothing about the person. Over time, however, you may get a feeling for how the person generally evaluates themselves.

You have 4 seconds for each answer; after that, the program will continue.

### ***Additional information on the profiles***

Names used: Emma, Hannah, Sophia, Marie, Lena, Julia

Ages used: 22, 24, 26, 27, 31, 33

### ***Task instructions for the self-ratings***

The following task instructions are translated from the German on-screen instructions:

Now, please evaluate yourself. You have 4 seconds for each answer; after that, the program will continue.

**Table S3** The 40 trait items used in the personality trait learning task

| Item number | German trait words used | Approximate English translation | Valence |
| --- | --- | --- | --- |
| **Factor 1: Neuroticism** | | |  |
| 1 | gelassen | composed | positive |
| 2 | locker | easy-going | positive |
| 3 | selbstständig | self-reliant | positive |
| 4 | souverän | confident | positive |
| 5 | ängstlich | anxious | negative |
| 6 | launisch | moody | negative |
| 7 | unentschlossen | Indecisive | negative |
| 8 | wehleidig | whiny | negative |
| **Factor 2: Extraversion** | | |  |
| 9 | enthusiastisch | enthusiastic | positive |
| 10 | lebenslustig | fun-loving | positive |
| 11 | schlagfertig | articulate | positive |
| 12 | tatkräftig | dynamic | positive |
| 13 | humorlos | humorless | negative |
| 14 | kalt | cold-hearted | negative |
| 15 | scheu | unassertive | negative |
| 16 | unnahbar | inapproachable | negative |
| **Factor 3: Openness** | | |  |
| 17 | kreativ | creative | positive |
| 18 | spontan | spontaneous | positive |
| 19 | tolerant | tolerant | positive |
| 20 | vielseitig | versatile | positive |
| 21 | engstirnig | narrow-minded | negative |
| 22 | träge | lazy | negative |
| 23 | voreingenommen | biased | negative |
| 24 | freundlich | friendly | negative |
| **Factor 4: Agreeableness** | | |  |
| 25 | großzügig | generous | positive |
| 26 | hilfsbereit | helpful | positive |
| 27 | höflich | polite | positive |
| 28 | arrogant | arrogant | negative |
| 29 | egoistisch | selfish | negative |
| 30 | hinterhältig | conniving | negative |
| 31 | stur | stubborn | negative |
| 32 | unsympathisch | unpleasant | negative |
| **Factor 5: Conscientiousness** | | |  |
| 33 | aufrichtig | honest | positive |
| 34 | bescheiden | modest | positive |
| 35 | fleißig | hard-working | positive |
| 36 | ordentlich | tidy | positive |
| 37 | leichtsinnig | foolhardy | negative |
| 38 | pedantisch | pedantic | negative |
| 39 | unpünktlich | tardy | negative |
| 40 | zwanghaft | obsessive | negative |

## **Inclusion and exclusion procedure**

We recruited female participants aged 18-40 who were fluent in German. Our preregistered criteria initially required individuals in the BPD group to meet at least five BPD criteria based on the IPDE interview. However, during recruitment, we found that about 20% of the participants with a formal BPD diagnosis did not meet this threshold. To ensure feasibility of data collection and enhance the clinical generalizability of our results, we expanded our inclusion criteria to include sub-threshold BPD participants meeting four IPDE criteria.

Exclusion criteria included severe neurological or physical illness, BMI < 15, severe visual impairments, cognitive disorders, a lifetime diagnosis of schizophrenia, schizoaffective or bipolar disorder, current narcissistic or avoidant personality disorder, acute suicidality, and being a psychology student (except first semester). Additional exclusions for BPD participants were moderate to severe alcohol or drug dependence and psychotropic medication use (except antidepressants or anxiolytics). However, due to challenges in recruiting enough eligible participants, we broadened our inclusion to individuals who received Quetiapine prescriptions to improve sleeping (up to 100 mg extended-release and 50 mg immediate-release) and on-demand medication for the BPD group. For controls, exclusions included current alcohol or drug dependence, any lifetime personality disorder, current or past psychiatric diagnoses or interventions (within the last 5 years), use of psychotropic medication, more than one BPD symptom (according to the IPDE interview), or current clinically relevant symptoms (according to the Mini-Dips).

We aimed to collect 30 valid datasets from women with BPD and 30 from an age- and gender-matched control group. A power analysis confirmed that this sample size was sufficient (see section Power analysis for details). As participants in this study also completed behavioral and fMRI experiments for a larger project mentioned above, we ultimately collected data from 100 participants (44 individuals with BPD and 56 controls).

Prior to analysis, 25 datasets (4 BPD, 21 controls) had to be excluded due to a programming error in the task code that we only noticed after collecting these datasets  (i.e., they did not receive the correct feedback due to a mistake in the randomization setup). We excluded an additional 10 participants in the BPD group for the following reasons: four did not meet the minimum requirement of four IPDE diagnostic criteria, two showed task comprehension difficulties, two had taken medications listed in our exclusion criteria, and two reported substance use before testing. Finally, we excluded four control participants who displayed clinically relevant symptoms according to the Mini-Dips during the diagnostic interview.

## **Computational models**

To model participants' learning behavior in the behavioral task, we employed eight models introduced by Frolichs et al. (3) and extended them into a hierarchical paradigm. These models capture the learning and generalization processes at three levels of granularity, forming three distinct model classes: **no learning**, **coarse granularity**, and **fine granularity**. Each of these classes includes at least one instance that utilizes **reference points**. These reference points represent average trait values, either reflecting average population values (population reference points) or an individual's own trait values (self-reference points).

### ***No learning models***

The first model class implements a learning-free approach, where predictions are derived from linear transformations of reference points. For each participant $j$ and trait $t$ with reference point $x_{t}$:

[
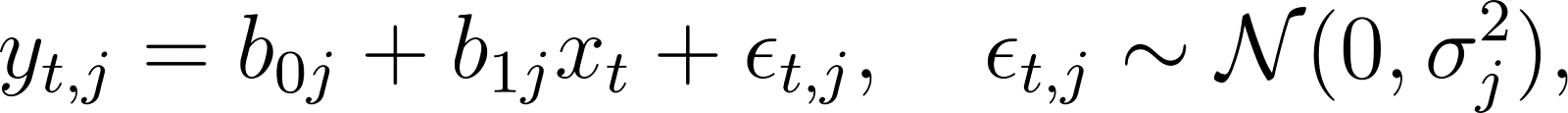
](https://www.codecogs.com/eqnedit.php?latex=y_%7Bt%2Cj%7D%20%3D%20b_%7B0j%7D%20%2B%20b_%7B1j%7Dx_t%20%2B%20%5Cepsilon_%7Bt%2Cj%7D%2C%20%5Cquad%20%5Cepsilon_%7Bt%2Cj%7D%20%5Csim%20%5Cmathcal%7BN%7D(0%2C%20%5Csigma_j%5E2)%2C#0)

with participant-level parameters:

[
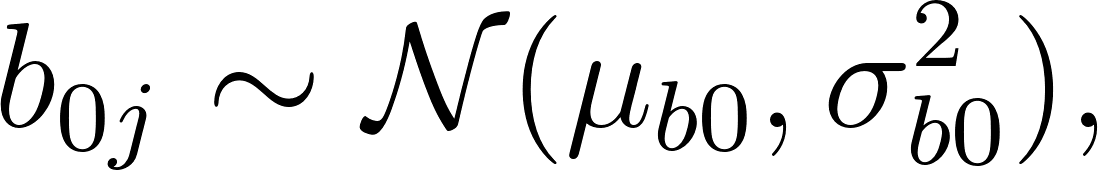
](https://www.codecogs.com/eqnedit.php?latex=b_%7B0j%7D%20%5Csim%20%5Cmathcal%7BN%7D(%5Cmu_%7Bb0%7D%2C%20%5Csigma_%7Bb0%7D%5E2)%2C#0)

[
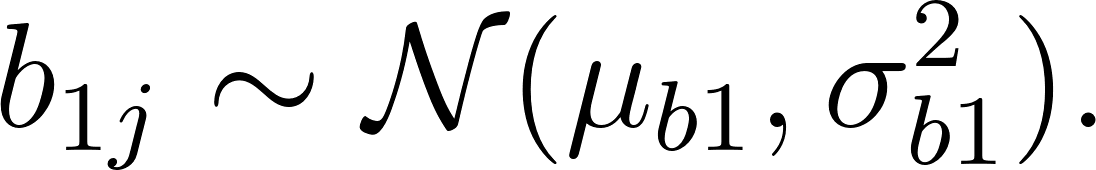
](https://www.codecogs.com/eqnedit.php?latex=b_%7B1j%7D%20%5Csim%20%5Cmathcal%7BN%7D(%5Cmu_%7Bb1%7D%2C%20%5Csigma_%7Bb1%7D%5E2).#0)

Since there are two types of reference points (population and self), this class consists of two models.

### ***Coarse granularity models***

The second class of models represents a coarse learning approach, where predictions are generalized across the Big Five personality dimensions within a Rescorla-Wagner learning framework. We denote the initial expectation for trait as [
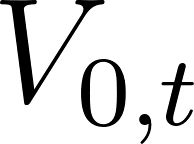
](https://www.codecogs.com/eqnedit.php?latex=V_%7B0%2Ct%7D#0), treating it as an item‐specific parameter drawn from a common distribution:

[
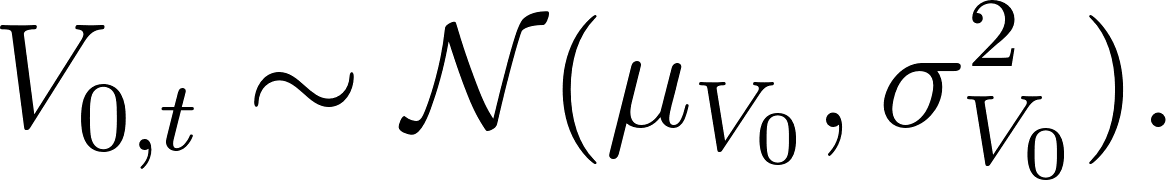
](https://www.codecogs.com/eqnedit.php?latex=V_%7B0%2Ct%7D%5Csim%5Cmathcal%7BN%7D(%5Cmu_%7BV_0%7D%2C%5Csigma_%7BV_0%7D%5E2).#0)

For each subsequent trial [
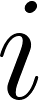
](https://www.codecogs.com/eqnedit.php?latex=i#0), the dimension $d$-specific expectation [
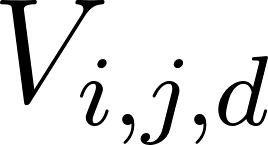
](https://www.codecogs.com/eqnedit.php?latex=V_%7Bi%2Cj%2Cd%7D#0) is then updated according to:

[
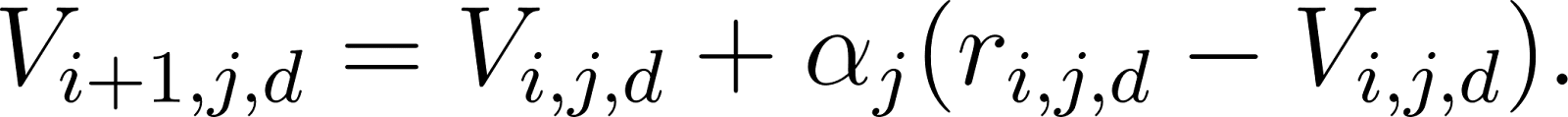
](https://www.codecogs.com/eqnedit.php?latex=V_%7Bi%20%2B%201%2Cj%2Cd%7D%3DV_%7Bi%2Cj%2Cd%7D%20%2B%20%5Calpha_j(r_%7Bi%2Cj%2Cd%7D%20-%20V_%7Bi%2Cj%2Cd%7D).#0)

Here, [
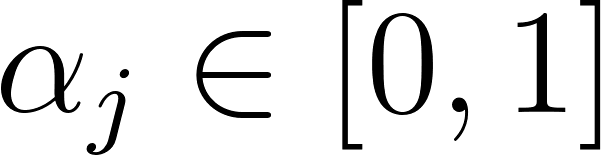
](https://www.codecogs.com/eqnedit.php?latex=%5Calpha_j%20%5Cin%20%5B0%2C1%5D#0) is a participant-specific learning rate that modulates the influence of the discrepancy between the feedback [
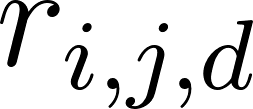
](https://www.codecogs.com/eqnedit.php?latex=r_%7Bi%2Cj%2Cd%7D#0) and the previous expectation [
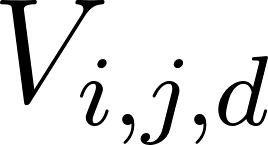
](https://www.codecogs.com/eqnedit.php?latex=V_%7Bi%2Cj%2Cd%7D#0) on the updated expectation. As with the no learning model class, parameters are modeled hierarchically. To ensure the learning rate respects its natural bounds, we employed the probit (normal CDF) transform:

[
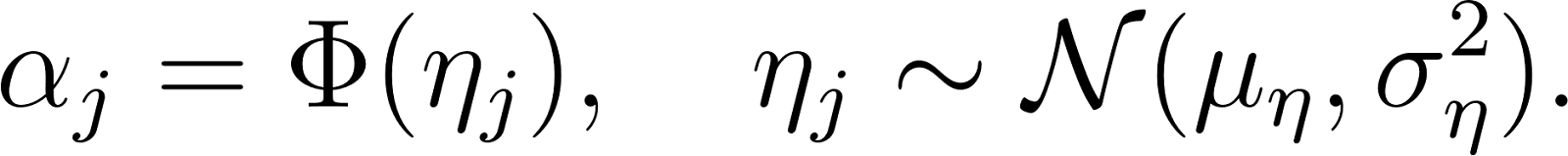
](https://www.codecogs.com/eqnedit.php?latex=%5Calpha_j%20%3D%20%5CPhi(%5Ceta_j)%2C%20%5Cquad%20%5Ceta_j%20%5Csim%20%5Cmathcal%7BN%7D(%5Cmu_%7B%5Ceta%7D%2C%20%5Csigma_%7B%5Ceta%7D%5E2).#0)

Given a participant's expectation [
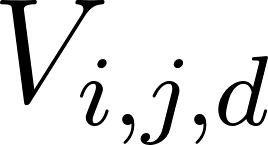
](https://www.codecogs.com/eqnedit.php?latex=V_%7Bi%2Cj%2Cd%7D#0), we estimate their prediction as:

[
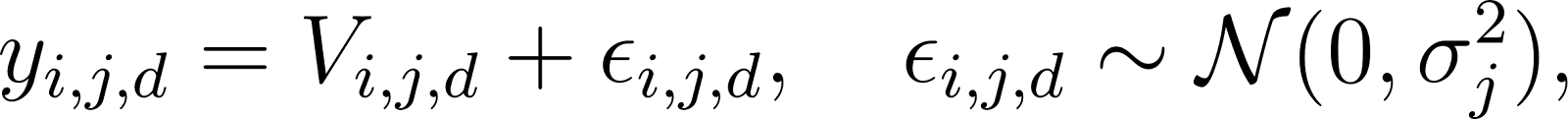
](https://www.codecogs.com/eqnedit.php?latex=y_%7Bi%2Cj%2Cd%7D%20%3DV_%7Bi%2Cj%2Cd%7D%20%2B%20%5Cepsilon_%7Bi%2Cj%2Cd%7D%2C%20%5Cquad%20%5Cepsilon_%7Bi%2Cj%2Cd%7D%20%5Csim%20%5Cmathcal%7BN%7D(0%2C%20%5Csigma_j%5E2)%2C#0)

where [
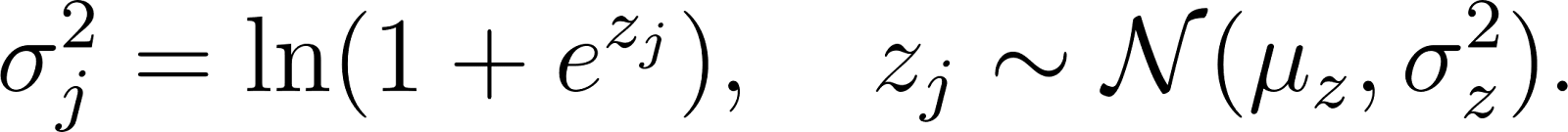
](https://www.codecogs.com/eqnedit.php?latex=%5Csigma_j%5E2%20%3D%20%5Cln(1%20%2B%20e%5E%7Bz_j%7D)%20%2C%20%5Cquad%20z_j%20%5Csim%20%5Cmathcal%7BN%7D(%5Cmu_z%2C%20%5Csigma_z%5E2).#0)

The addition of the error term [
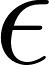
](https://www.codecogs.com/eqnedit.php?latex=%5Cepsilon#0) and its associated participant-specific variance [
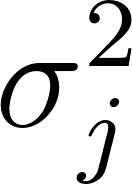
](https://www.codecogs.com/eqnedit.php?latex=%5Csigma_j%5E2#0) models stochastic deviations from the otherwise deterministic learning rule.

### ***Fine granularity models***

Finally, the third model class addresses the generalization process at the finest level by modeling separate expectations [
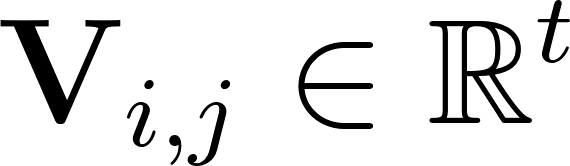
](https://www.codecogs.com/eqnedit.php?latex=%5Cmathbf%7BV%7D_%7Bi%2Cj%7D%20%5Cin%20%5Cmathbb%7BR%7D%5Et#0) for each trait [
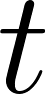
](https://www.codecogs.com/eqnedit.php?latex=t#0). These expectations are updated using a similarity matrix [
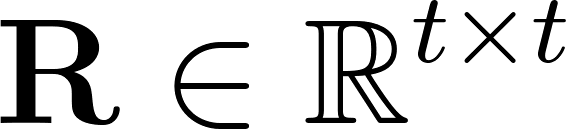
](https://www.codecogs.com/eqnedit.php?latex=%5Cmathbf%7BR%7D%20%5Cin%20%5Cmathbb%7BR%7D%5E%7Bt%20%5Ctimes%20t%7D#0), which was obtained by computing pairwise correlations from data in previous studies (2,4):

[
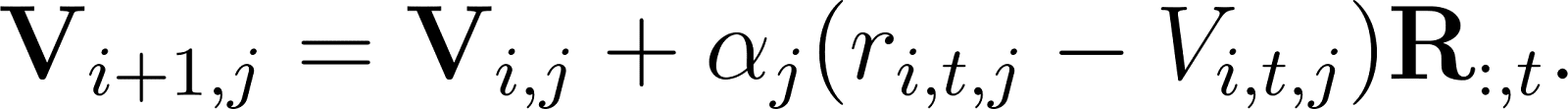
](https://www.codecogs.com/eqnedit.php?latex=%5Cmathbf%7BV%7D_%7Bi%2B1%2Cj%7D%3D%5Cmathbf%7BV%7D_%7Bi%2Cj%7D%20%2B%20%5Calpha_j(r_%7Bi%2Ct%2Cj%7D%20-%20V_%7Bi%2Ct%2Cj%7D)%5Cmathbf%7BR%7D_%7B%3A%2Ct%7D.#0)

Both the coarse and the fine granularity model class include models that make use of the population or self specific reference points [
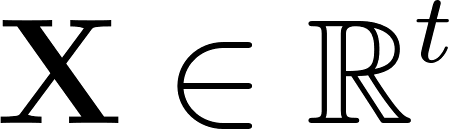
](https://www.codecogs.com/eqnedit.php?latex=%5Cmathbf%7BX%7D%20%5Cin%20%5Cmathbb%7BR%7D%5Et#0). For the fine granularity models, the predictions are made as:

[
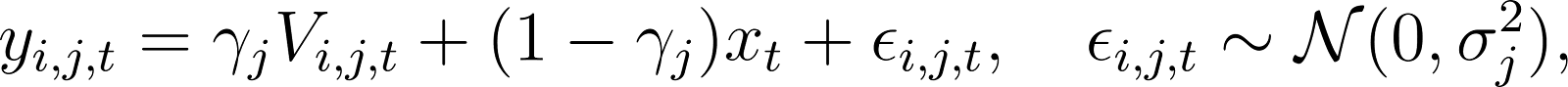
](https://www.codecogs.com/eqnedit.php?latex=y_%7Bi%2Cj%2Ct%7D%3D%5Cgamma_j%20V_%7Bi%2Cj%2Ct%7D%20%2B%20(1-%5Cgamma_j)x_t%20%2B%20%5Cepsilon_%7Bi%2Cj%2Ct%7D%2C%20%5Cquad%20%5Cepsilon_%7Bi%2Cj%2Ct%7D%20%5Csim%20%5Cmathcal%7BN%7D(0%2C%5Csigma_j%5E2)%2C#0)

where [
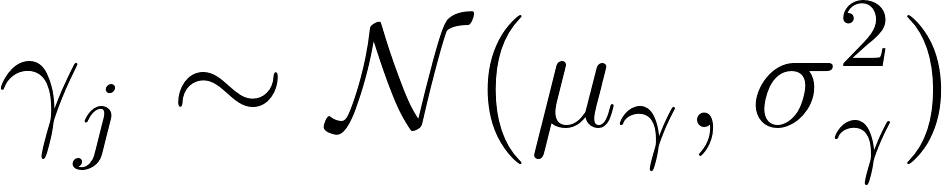
](https://www.codecogs.com/eqnedit.php?latex=%5Cgamma_j%20%5Csim%20%5Cmathcal%7BN%7D(%5Cmu_%7B%5Cgamma%7D%2C%20%5Csigma_%7B%5Cgamma%7D%5E2)#0) is a participant-specific weighting parameter that modulates the influence of expectations versus reference points on the predictions. Predictions for the coarse granularity models are constructed similarly, using personality dimension [
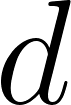
](https://www.codecogs.com/eqnedit.php?latex=d#0) instead of trait [
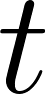
](https://www.codecogs.com/eqnedit.php?latex=t#0). Thus, both the coarse and the final granularity classes contain three models each (none, self, or population reference points).

## **Model fitting**

All models were fitted using Stan (5) and the rstan interface (6). Stan is a probabilistic programming language designed for Bayesian statistical inference, utilizing techniques such as Hamiltonian Markov Chain Monte Carlo sampling. The framework lends itself well to hierarchical estimation which often surpasses non-hierarchical methods in accuracy (7,8). To obtain parameter estimates, we ran the NUTS algorithm with eight MCMC chains and 2000 iterations each, ensuring that all diagnostic criteria remained within ranges desired for accurate inference (9). Given that the computational load of full Hamiltonian MCMC runs is prohibitively expensive in combination with cross-validation, we used Stan’s variational algorithm for approximate posterior sampling during model comparison.

## **Model comparison**

All models include a participant-specific residual variance parameter [
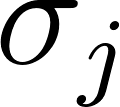
](https://www.codecogs.com/eqnedit.php?latex=%5Csigma_j#0) that captures the variability in predictions for each participant, accounting for individual differences not explained by the model. As a byproduct, this facilitates the calculation of the model likelihood [
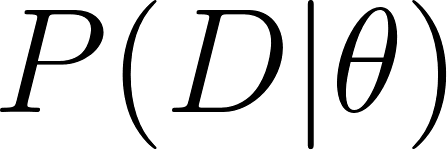
](https://www.codecogs.com/eqnedit.php?latex=P(D%7C%5Ctheta)#0) via the normal distribution's log probability density function:

[
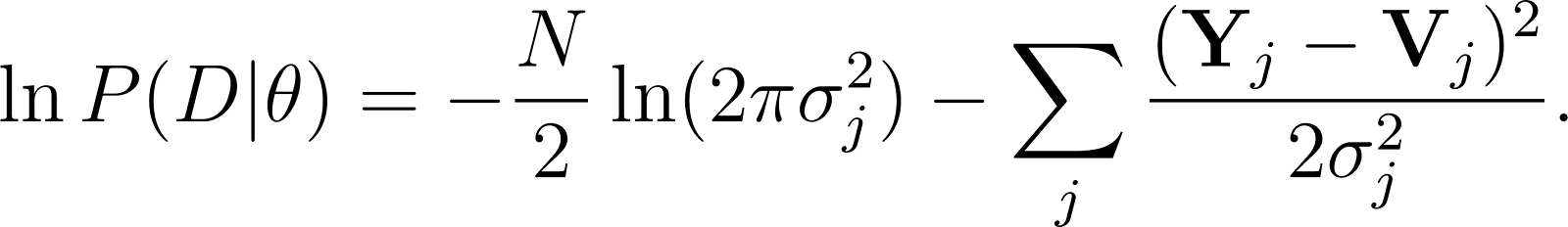
](https://www.codecogs.com/eqnedit.php?latex=%5Cln%20P(D%7C%5Ctheta)%20%3D%20-%5Cfrac%7BN%7D%7B2%7D%5Cln(2%5Cpi%5Csigma_j%5E2)%20-%20%5Csum_%7Bj%7D%5Cfrac%7B(%5Cmathbf%7BY%7D_j-%5Cmathbf%7BV%7D_j)%5E2%7D%7B2%5Csigma_j%5E2%7D.#0)

We employed a Bayesian group-[
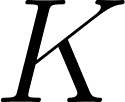
](https://www.codecogs.com/eqnedit.php?latex=K#0)-fold cross-validation scheme to estimate the out of sample likelihood on unseen participants [
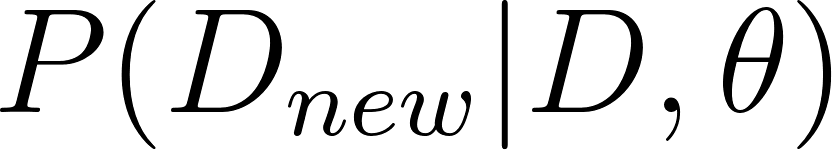
](https://www.codecogs.com/eqnedit.php?latex=P(D_%7Bnew%7D%7CD%2C%20%5Ctheta)#0). In the case of Bayesian models, this likelihood takes the form of the expected predictive density over the posterior draws based on the training folds. Specifically, after dividing the participants into [
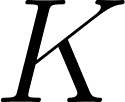
](https://www.codecogs.com/eqnedit.php?latex=K#0) disjoint subsets (folds) of approximately equal size, each model is trained on [
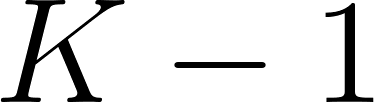
](https://www.codecogs.com/eqnedit.php?latex=K-1#0) folds, leaving out one fold for validation. This process is repeated [
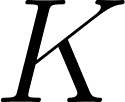
](https://www.codecogs.com/eqnedit.php?latex=K#0) times so that each participant subset serves as a hold-out set exactly once. For Bayesian models, let [
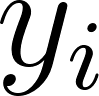
](https://www.codecogs.com/eqnedit.php?latex=y_%7Bi%7D#0) be an outcome for participant [
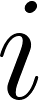
](https://www.codecogs.com/eqnedit.php?latex=i#0) in the held-out fold and [
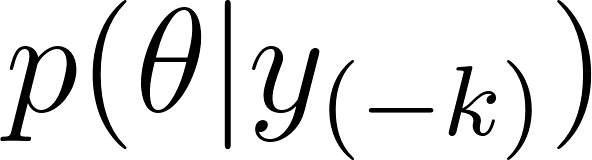
](https://www.codecogs.com/eqnedit.php?latex=p(%5Ctheta%20%7C%20y_%7B(-k)%7D)#0) denote the posterior distribution of the parameters [
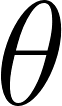
](https://www.codecogs.com/eqnedit.php?latex=%5Ctheta#0) based on the training folds [
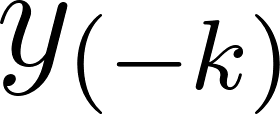
](https://www.codecogs.com/eqnedit.php?latex=y_%7B(-k)%7D#0). The predictive likelihood for each held-out data point is then given by the posterior predictive integral:

[
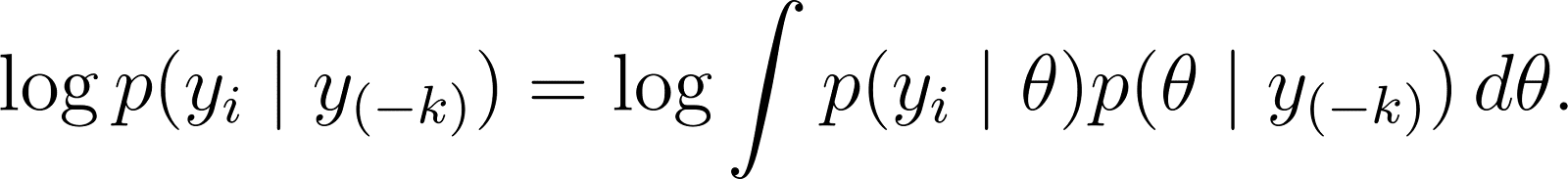
](https://www.codecogs.com/eqnedit.php?latex=%5Clog%20p(y_i%20%5Cmid%20y_%7B(-k)%7D)%20%3D%20%5Clog%20%5Cint%20p(y_i%20%5Cmid%20%5Ctheta)p(%5Ctheta%20%5Cmid%20y_%7B(-k)%7D)%20%5C%2C%20d%5Ctheta.#0)

Because [
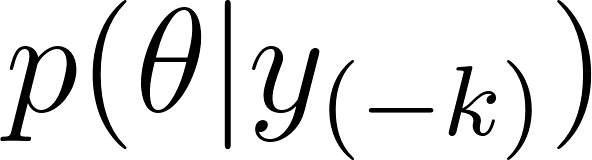
](https://www.codecogs.com/eqnedit.php?latex=p(%5Ctheta%20%7C%20y_%7B(-k)%7D)#0) is available only through Monte Carlo draws from the posterior, we approximate the integral using [
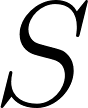
](https://www.codecogs.com/eqnedit.php?latex=S#0) samples [
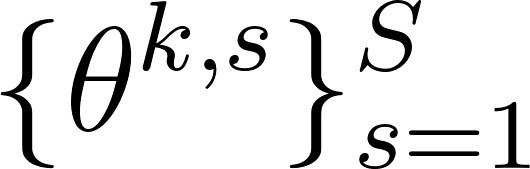
](https://www.codecogs.com/eqnedit.php?latex=%5C%7B%5Ctheta%5E%7Bk%2Cs%7D%5C%7D_%7Bs%3D1%7D%5ES#0) drawn from the posterior of the [
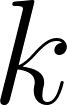
](https://www.codecogs.com/eqnedit.php?latex=k#0)-th training fold:

[
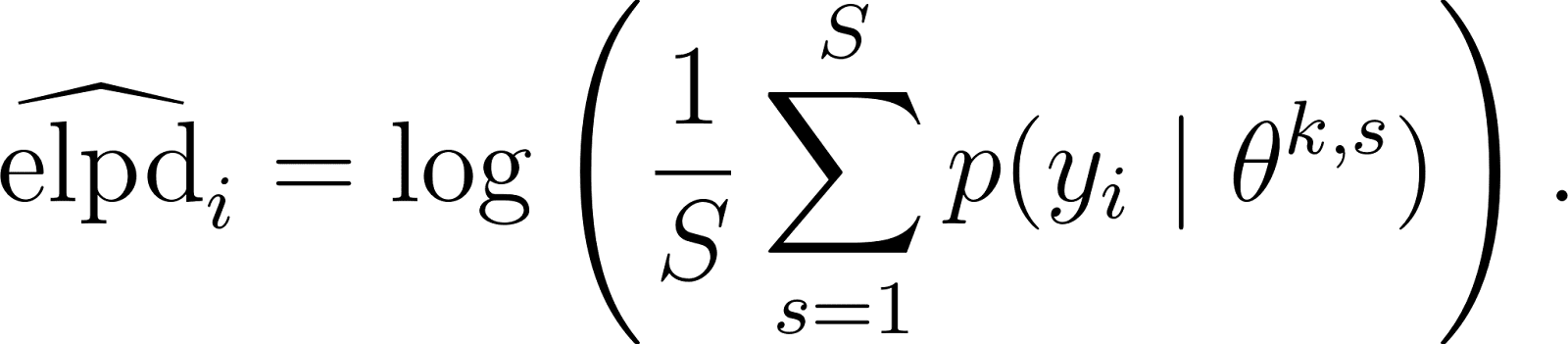
](https://www.codecogs.com/eqnedit.php?latex=%5Cwidehat%7B%5Ctext%7Belpd%7D%7D_i%20%3D%20%5Clog%20%5Cleft(%20%5Cfrac%7B1%7D%7BS%7D%5Csum_%7Bs%3D1%7D%5ES%20p(y_i%20%5Cmid%20%5Ctheta%5E%7Bk%2Cs%7D)%20%5Cright).#0)

Summing across all held-out points from all folds, we obtain an estimate of the expected log predictive density:

[
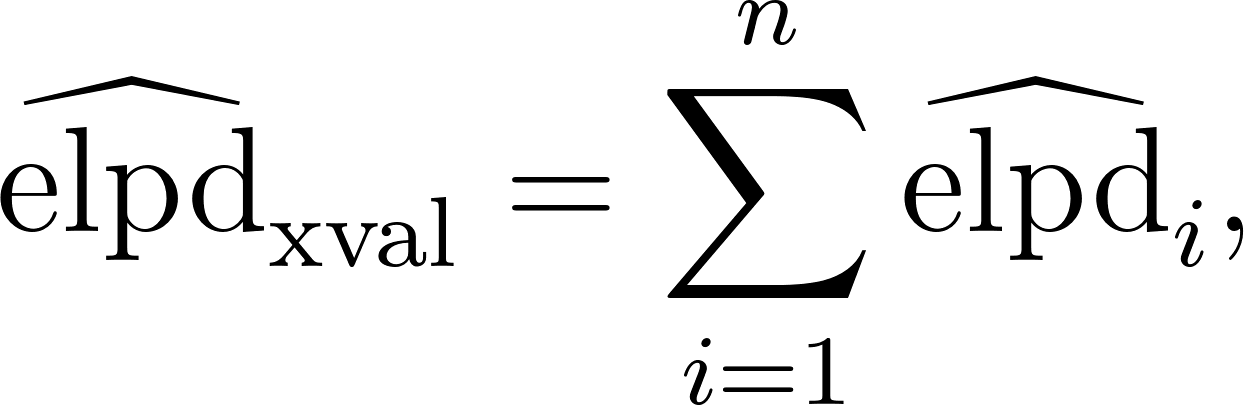
](https://www.codecogs.com/eqnedit.php?latex=%5Cwidehat%7B%5Ctext%7Belpd%7D%7D_%7B%5Ctext%7Bxval%7D%7D%20%3D%20%5Csum_%7Bi%3D1%7D%5En%20%5Cwidehat%7B%5Ctext%7Belpd%7D%7D_i%2C#0)

where [
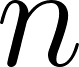
](https://www.codecogs.com/eqnedit.php?latex=n#0) is the total number of data points. The model with the highest elpd score is considered the best fit to the data. To get an idea of the uncertainty associated with the ranking of models based on their elpd estimates, we compute standard errors of their differences. Although no formal decision boundary akin to a frequentist significance level exists, performance differences between models [
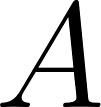
](https://www.codecogs.com/eqnedit.php?latex=A#0) and [
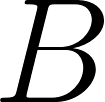
](https://www.codecogs.com/eqnedit.php?latex=B#0) are sometimes considered “significant” when [
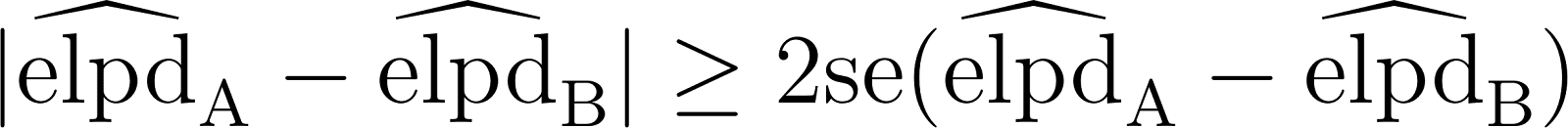
](https://www.codecogs.com/eqnedit.php?latex=%7C%20%5Cwidehat%7B%5Ctext%7Belpd%7D%7D_%7B%5Ctext%7BA%7D%7D%20%20-%20%5Cwidehat%7B%5Ctext%7Belpd%7D%7D_%7B%5Ctext%7BB%7D%7D%20%7C%20%20%5Cge%202%20%5Ctext%7Bse%7D(%20%5Cwidehat%7B%5Ctext%7Belpd%7D%7D_%7B%5Ctext%7BA%7D%7D%20%20-%20%5Cwidehat%7B%5Ctext%7Belpd%7D%7D_%7B%5Ctext%7BB%7D%7D%20)#0), which is approximately equivalent to performing a conventional significance test (10).

For our study, we set the number of holdout folds, [
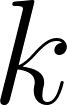
](https://www.codecogs.com/eqnedit.php?latex=k#0), to the number of participants in the data. This effectively performs leave-one-participant-out cross-validation, which is simultaneously the most accurate and computationally expensive approach available for our data.

# **Supplementary results**

## **Self-ratings on trait adjectives**

**Table S4** LME model on the effect of Group on Self-Ratings

| *Predictors* | *b* | *SE* | *95% CI* | *t* | *p* | *df* |
| --- | --- | --- | --- | --- | --- | --- |
| (Intercept) | 5.44 | 0.14 | 5.16 – 5.73 | 37.57 | **<0.001** | 61.63 |
| Group [CON] | 0.77 | 0.11 | 0.56 – 0.99 | 7.16 | **<0.001** | 58.76 |
| **Random Effects** | | | | | | |
| σ^2^ | 2.19 | | | | | |
| τ_00_ _sub_ | 0.12 | | | | | |
| τ_00_ _item_ | 0.60 | | | | | |
| ICC | 0.25 | | | | | |
| N _sub_ | 61 | | | | | |
| N _item_ | 40 | | | | | |
| Observations | 2406 | | | | | |
| Marginal R^2^ / Conditional R^2^ | 0.049 / 0.285 | | | | | |

Note. Significant results in bold font

**Table S5** Type III ANOVA on the effect of Group on Self-Ratings

| *Predictors* | *Sum of Squares* | *Mean Square* | *df_1_* | *df₂* | *F* | *p* |
| --- | --- | --- | --- | --- | --- | --- |
| Group | 112.45 | 112.45 | 1 | 58.76 | 51.30 | **< 0.001** |

Note. *df₁* = numerator degrees of freedom; *df₂* = denominator degrees of freedom; Significant results in bold font

**Table S6** LME model on the effect of Group, Valence, and their interaction on Self-Ratings

| *Predictors* | *b* | *SE* | *95% CI* | *t* | *p* | *df* |
| --- | --- | --- | --- | --- | --- | --- |
| (Intercept) | 3.89 | 0.19 | 3.52 – 4.27 | 20.77 | **<0.001** | 48.72 |
| Group [CON] | -1.05 | 0.11 | -1.27 – -0.84 | -9.57 | **<0.001** | 120.51 |
| Valence [positive] | 1.89 | 0.26 | 1.37 – 2.41 | 7.39 | **<0.001** | 42.87 |
| Group [CON] × Valence [positive] | 1.54 | 0.12 | 1.31 – 1.78 | 12.71 | **<0.001** | 2305.41 |
| **Random Effects** | | | | | | |
| σ^2^ | 2.22 | | | | | |
| τ_00_ _sub_ | 0.07 | | | | | |
| τ_00_ _item_ | 0.58 | | | | | |
| ICC | 0.23 | | | | | |
| N _sub_ | 61 | | | | | |
| N _item_ | 40 | | | | | |
| Observations | 2406 | | | | | |
| Marginal R^2^ / Conditional R^2^ | 0.406 / 0.541 | | | | | |

Note. Significant results in bold font

**Table S7** Type III ANOVA on the effect of Group, Valence, and their interaction on Self-Ratings

| *Predictors* | *Sum of Squares* | *Mean Square* | *df_1_* | *df₂* | *F* | *p* |
| --- | --- | --- | --- | --- | --- | --- |
| Group | 20.88 | 20.88 | 1 | 58.92 | 9.40 | **0.003** |
| Valence | 255.35 | 255.35 | 1 | 38.00 | 115.02 | **< 0.001** |
| Group × Valence | 358.40 | 358.40 | 1 | 2,305.41 | 161.43 | **< 0.001** |

Note. *df₁* = numerator degrees of freedom; *df₂* = denominator degrees of freedom; Significant results in bold font

**Table S8** EMMs Contrasts of Group Differences Within Each Valence

| *Valence* | *Contrast* | *Estimate* | *SE* | *df* | *CI(LL)* | *CI(UL)* | *t* | *p* |
| --- | --- | --- | --- | --- | --- | --- | --- | --- |
| Negative | Group BPD - Group CON | 1.054 | 0.11 | 120.644 | 0.836 | 1.272 | 9.574 | **< 0.001** |
| Positive | Group BPD - Group CON | -0.490 | 0.11 | 121.671 | -0.709 | -0.272 | -4.441 | **< 0.001** |

Note. Contrasts reflect differences in predicted values of Self-Ratings between Groups within each Valence. Degrees-of-freedom method: Kenward-Roger. *CI*: 95% Confidence Intervals with lower limits (*LL*) and upper limits (*UL*). Significant results in bold font

## **Self-ratings on personality inventories**

### ***NEO-FFI***

**Table S9** Multivariate Analysis of Variance (MANOVA) on the effect of Group on the NEO-FFI personality factors

| *Effect* | *DF* | *Pillai‘s Trace* | *F (approx..)* | *df1* | *df2* | *p* |
| --- | --- | --- | --- | --- | --- | --- |
| (Intercept) | 1.00 | 0.99 | 1,431.04 | 5.00 | 55.00 | **<0.001** |
| Group | 1.00 | 0.77 | 36.10 | 5.00 | 55.00 | **<0.001** |
| Residuals | 59.00 |  |  |  |  |  |

DF = degrees of freedom for the terms; df1 = degrees of freedom in the model; df2 = degrees of freedom associated with the model errors; Significant results in bold font

**Table S10** Post-hoc Welch’s t-tests comparing BPD and control groups on NEO-FFI personality factors

|  | BPD | | CON | |  |  |  |  |  |  |  |
| --- | --- | --- | --- | --- | --- | --- | --- | --- | --- | --- | --- |
| Factor | *M* | *SD* | *M* | *SD* | *t* | *DF* | *CI (LL)* | *CI (UL)* | *d* | *p* | *p (fdr)* |
| N | 3.09 | 0.44 | 1.55 | 0.52 | 12.54 | 57.90 | 1.30 | 1.79 | 3.20 | **<0.001** | **<0.001** |
| E | 1.93 | 0.63 | 2.33 | 0.66 | -2.43 | 59.00 | -0.73 | -0.07 | -0.62 | **0.018** | **0.022** |
| O | 2.49 | 0.56 | 2.31 | 0.46 | 1.33 | 55.80 | -0.09 | 0.44 | 0.34 | 0.190 | 0.190 |
| A | 2.39 | 0.49 | 2.98 | 0.44 | -4.88 | 58.00 | -0.82 | -0.34 | -1.25 | **<0.001** | **<0.001** |
| C | 2.21 | 0.55 | 2.94 | 0.61 | -4.92 | 58.60 | -1.03 | -0.43 | -1.26 | **<0.001** | **<0.001** |

Factors: N = Neuroticism, E = Extraversion, O = Openness, A= Agreeableness, C = Conscientiousness; CI = 95% confidence interval; LL = lower limit; UL = upper limit; d = Cohen’s d; p (fdr) = p-value adjusted for false discovery rate; Significant results in bold font

### ***PID-5-BF***

**Table S11** Multivariate Analysis of Variance (MANOVA) on the effect of Group on the PID-5-BF domains

| *Effect* | *DF* | *Pillai‘s Trace* | *F (approx..)* | *df1* | *df2* | *p* |
| --- | --- | --- | --- | --- | --- | --- |
| (Intercept) | 1.00 | 0.92 | 125.79 | 5.00 | 55.00 | **<0.001** |
| Group | 1.00 | 0.68 | 23.86 | 5.00 | 55.00 | **<0.001** |
| Residuals | 59.00 |  |  |  |  |  |

DF = degrees of freedom for the terms; df1 = degrees of freedom in the model; df2 = degrees of freedom associated with the model errors; Significant results in bold font

**Table S12** Post-hoc Welch’s t-tests comparing BPD and control groups on the PID-5-BF domains

|  | BPD | | CON | |  |  |  |  |  |  |  |
| --- | --- | --- | --- | --- | --- | --- | --- | --- | --- | --- | --- |
| Factor | *M* | *SD* | *M* | *SD* | *t* | *DF* | *CI (LL)* | *CI (UL)* | *d* | *p* | *p (fdr)* |
| Negative Affect | 10.50 | 2.01 | 4.61 | 2.79 | 9.48 | 54.60 | 4.64 | 7.13 | 2.41 | **<0.001** | **<0.001** |
| Detachment | 6.63 | 2.58 | 2.87 | 2.47 | 5.81 | 58.70 | 2.47 | 5.06 | 1.49 | **<0.001** | **<0.001** |
| Antagonism | 4.23 | 3.50 | 1.39 | 1.71 | 4.02 | 41.70 | 1.42 | 4.28 | 1.04 | **<0.001** | **<0.001** |
| Disinhibition | 8.13 | 3.08 | 2.65 | 2.47 | 7.66 | 55.50 | 4.05 | 6.92 | 1.97 | **<0.001** | **<0.001** |
| Psychoticism | 7.70 | 3.63 | 1.84 | 1.73 | 8.00 | 41.30 | 4.38 | 7.34 | 2.07 | **<0.001** | **<0.001** |

CI = 95% confidence interval; LL = lower limit; UL = upper limit; d = Cohen’s d; p (fdr) = p-value adjusted for false discovery rate; Significant results in bold font

## **Other-ratings**

**Table S1****3** LME model on the effect of Group, Valence, and their interaction on Other-Ratings

| *Predictors* | *b* | *SE* | *95% CI* | *t* | *p* | *df* |
| --- | --- | --- | --- | --- | --- | --- |
| (Intercept) | 3.78 | 0.10 | 3.58 – 3.97 | 38.26 | **<0.001** | 65.03 |
| Group [CON] | -0.14 | 0.07 | -0.29 – 0.01 | -1.83 | 0.071 | 70.82 |
| Valence [positive] | 1.88 | 0.12 | 1.64 – 2.13 | 15.48 | **<0.001** | 40.69 |
| Group [CON] × Valence [positive] | 0.23 | 0.04 | 0.14 – 0.31 | 5.20 | **<0.001** | 13958.68 |
| **Random Effects** | | | | | | |
| σ^2^ | 1.69 | | | | | |
| τ_00_ _sub_ | 0.07 | | | | | |
| τ_00_ _item_ | 0.14 | | | | | |
| ICC | 0.11 | | | | | |
| N _sub_ | 61 | | | | | |
| N _item_ | 40 | | | | | |
| Observations | 14059 | | | | | |
| Marginal R^2^ / Conditional R^2^ | 0.346 / 0.418 | | | | | |

Note. Significant results in bold font

**Table S14** Type III ANOVA on the effect of Group, Valence, and their interaction on Other-Ratings

| *Predictors* | *Sum of Squares* | *Mean Square* | *df_1_* | *df₂* | *F* | *p* |
| --- | --- | --- | --- | --- | --- | --- |
| Group | 0.17 | 0.17 | 1 | 58.96 | 0.10 | 0.750 |
| Valence | 471.03 | 471.03 | 1 | 38.02 | 278.99 | **< 0.001** |
| Group × Valence | 45.72 | 45.72 | 1 | 13,958.68 | 27.08 | **< 0.001** |

Note. *df₁* = numerator degrees of freedom; *df₂* = denominator degrees of freedom; Significant results in bold font

**Table S15** EMMs Contrasts of Group Differences Within Each Valence

| *Valence* | *Contrast* | *Estimate* | *SE* | *z* | *p* |
| --- | --- | --- | --- | --- | --- |
| Negative | Group BPD - Group CON | 0.137 | 0.075 | 1.835 | 0.067 |
| Positive | Group BPD - Group CON | -0.091 | 0.075 | -1.224 | 0.221 |

**Note.** Contrasts reflect differences in predicted values of Other-Ratings between Groups within each Valence. Degrees-of-freedom method: asymptotic. Df values were not included due to high memory and computation load. Significant results in bold font

**Table S1****6** LME model on the effect of Group, Profile Type, and their interaction on Other-Ratings

| *Predictors* | *b* | *SE* | *95% CI* | *t* | *p* | *df* |
| --- | --- | --- | --- | --- | --- | --- |
| (Intercept) | 5.41 | 0.09 | 5.24 – 5.58 | 62.64 | **<0.001** | 82.50 |
| Group [CON] | -0.01 | 0.08 | -0.16 – 0.14 | -0.17 | 0.863 | 69.90 |
| Profile Type [CON] | 0.07 | 0.03 | 0.01 – 0.13 | 2.30 | **0.022** | 13963.05 |
| Group [CON] × Profile Type [CON] | 0.26 | 0.04 | 0.17 – 0.34 | 5.86 | **<0.001** | 13964.15 |
| **Random Effects** | | | | | | |
| σ^2^ | 1.67 | | | | | |
| τ_00_ _sub_ | 0.07 | | | | | |
| τ_00_ _item_ | 0.18 | | | | | |
| ICC | 0.13 | | | | | |
| N _sub_ | 61 | | | | | |
| N _item_ | 40 | | | | | |
| Observations | 14059 | | | | | |
| Marginal R^2^ / Conditional R^2^ | 0.009 / 0.140 | | | | | |

Note. Significant results in bold font

**Table S17** Type III ANOVA on the effect of Group, Profile Type, and their interaction on Other-Ratings

| *Predictors* | *Sum of Squares* | *Mean Square* | *df_1_* | *df₂* | *F* | *p* |
| --- | --- | --- | --- | --- | --- | --- |
| Group | 4.13 | 4.13 | 1 | 59.08 | 2.47 | 0.121 |
| Profile Type | 139.23 | 139.23 | 1 | 13,964.49 | 83.36 | **< 0.001** |
| Group × Profile Type | 57.39 | 57.39 | 1 | 13,964.15 | 34.36 | **< 0.001** |

Note. *df₁* = numerator degrees of freedom; *df₂* = denominator degrees of freedom; Significant results in bold font

**Table S18** EMMs Contrasts of Group Differences Within Each Profile Type

| *Profile Type* | *Contrast* | *Estimate* | *SE* | *z* | *p* |  |
| --- | --- | --- | --- | --- | --- | --- |
| BPD | Group BPD - Group CON | 0.013 | 0.076 | 0.173 | 0.863 |  |
| CON | Group BPD - Group CON | -0.243 | 0.076 | -3.185 | **0.001** |  |

Note. Contrasts reflect differences in predicted values of Other-Ratings between Groups within each Profile Type. Degrees-of-freedom method: asymptotic. Df values were not included due to high memory and computation load. Significant results in bold font

## **Prediction errors**

**Table S1****9** LME model on the effect of Group, Profile Type, and their interaction on PEs

| *Predictors* | *b* | *SE* | *95% CI* | *t* | *p* | *df* |
| --- | --- | --- | --- | --- | --- | --- |
| (Intercept) | 1.52 | 0.06 | 1.40 – 1.63 | 25.55 | **<0.001** | 87.10 |
| Group [CON] | 0.00 | 0.05 | -0.11 – 0.11 | 0.06 | 0.956 | 77.25 |
| Profile Type [CON] | -0.09 | 0.03 | -0.15 – -0.04 | -3.32 | **0.001** | 13966.57 |
| Group [CON] × Profile Type [CON] | -0.17 | 0.04 | -0.24 – -0.09 | -4.28 | **<0.001** | 13968.22 |
| **Random Effects** | | | | | | |
| σ^2^ | 1.33 | | | | | |
| τ_00_ _sub_ | 0.03 | | | | | |
| τ_00_ _item_ | 0.08 | | | | | |
| ICC | 0.08 | | | | | |
| N _sub_ | 61 | | | | | |
| N _item_ | 40 | | | | | |
| Observations | 14059 | | | | | |
| Marginal R^2^ / Conditional R^2^ | 0.008 / 0.086 | | | | | |

Note. Significant results in bold font

**Table S20** Type III ANOVA on the effect of Group, Profile Type, and their interaction on PEs

| *Predictors* | *Sum of Squares* | *Mean Square* | *df_1_* | *df₂* | *F* | *p* |
| --- | --- | --- | --- | --- | --- | --- |
| Group | 3.31 | 3.31 | 1 | 59.15 | 2.48 | 0.120 |
| Profile Type | 108.53 | 108.53 | 1 | 13,968.82 | 81.30 | **< 0.001** |
| Group × Profile Type | 24.50 | 24.50 | 1 | 13,968.22 | 18.36 | **< 0.001** |

Note. *df₁* = numerator degrees of freedom; *df₂* = denominator degrees of freedom; Significant results in bold font

**Table S21** EMMs Contrasts of Group Differences Within Each Profile Type

| *Profile Type* | *Contrast* | *Estimate* | *SE* | *z* | *p* |
| --- | --- | --- | --- | --- | --- |
| BPD | Group BPD - Group CON | -0.003 | 0.055 | -0.056 | 0.956 |
| CON | Group BPD - Group CON | 0.164 | 0.055 | 2.996 | **0.003** |

**Note.** Contrasts reflect differences in predicted values of PEs between Groups within each Profile Type. Degrees-of-freedom method: asymptotic. Df values were not included due to high memory and computation load. Significant results in bold font

**Table S22** EMMs Contrasts of Profile Type Differences Within Each Group

| *Group* | *Contrast* | *Estimate* | *SE* | *z* | *p* |
| --- | --- | --- | --- | --- | --- |
| BPD | Profile Type BPD - Profile Type CON | 0.092 | 0.028 | 3.324 | **0.001** |
| CON | Profile Type BPD - Profile Type CON | 0.260 | 0.027 | 9.473 | **<0.001** |

**Note.** Contrasts reflect differences in predicted values of PEs between Profile Type within each Group. Degrees-of-freedom method: asymptotic. Df values were not included due to high memory and computation load. Significant results in bold font

**Table S23** LME model on the effect of Trial on PEs

| *Predictors* | *b* | *SE* | *95% CI* | *t* | *p* | *df* |
| --- | --- | --- | --- | --- | --- | --- |
| (Intercept) | 1.49 | 0.05 | 1.38 – 1.60 | 27.13 | **<0.001** | 76.34 |
| Trial | -0.00 | 0.00 | -0.00 – -0.00 | -3.10 | **0.002** | 13875.47 |
| **Random Effects** | | | | | | |
| σ^2^ | 1.34 | | | | | |
| τ_00_ _sub_ | 0.03 | | | | | |
| τ_00_ _item_ | 0.08 | | | | | |
| ICC | 0.08 | | | | | |
| N _sub_ | 61 | | | | | |
| N _item_ | 40 | | | | | |
| Observations | 14059 | | | | | |
| Marginal R^2^ / Conditional R^2^ | 0.001 / 0.079 | | | | | |

Note. Significant results in bold font

**Table S24** Type III ANOVA on the effect of Trial on PEs

| *Predictors* | *Sum of Squares* | *Mean Square* | *df_1_* | *df₂* | *F* | *p* |
| --- | --- | --- | --- | --- | --- | --- |
| Trial | 12.94 | 12.94 | 1 | 13,875.47 | 9.63 | **0.002** |

Note. *df₁* = numerator degrees of freedom; *df₂* = denominator degrees of freedom; Significant results in bold font

**Table S 25** LME model on the effect of Trial, Group, Profile Type, and their interaction on PEs

| Predictors | b | SE | 95% CI | t | p | df |
| --- | --- | --- | --- | --- | --- | --- |
| (Intercept) | 1.54 | 0.07 | 1.40 – 1.67 | 22.16 | **<0.001** | 164.82 |
| Trial | -0.00 | 0.00 | -0.00 – 0.00 | -0.61 | 0.540 | 13969.69 |
| Group [CON] | 0.02 | 0.07 | -0.12 – 0.17 | 0.32 | 0.748 | 246.21 |
| Profile Type [CON] | -0.04 | 0.06 | -0.15 – 0.08 | -0.62 | 0.534 | 13988.44 |
| Trial × Group [CON] | -0.00 | 0.00 | -0.01 – 0.00 | -0.42 | 0.674 | 13953.68 |
| Trial × profile Type [CON] | -0.00 | 0.00 | -0.01 – 0.00 | -1.05 | 0.292 | 13967.61 |
| Group [CON] × Profile Type [CON] | -0.18 | 0.08 | -0.34 – -0.02 | -2.23 | **0.026** | 13956.60 |
| Trial × Group [CON] × Profile Type [CON] | 0.00 | 0.00 | -0.01 – 0.01 | 0.17 | 0.862 | 13954.14 |
| **Random Effects** | | | | | | |
| σ^2^ | 1.33 | | | | | |
| τ_00_ _sub_ | 0.03 | | | | | |
| τ_00_ _item_ | 0.08 | | | | | |
| ICC | 0.08 | | | | | |
| N _sub_ | 61 | | | | | |
| N _item_ | 40 | | | | | |
| Observations | 14059 | | | | | |
| Marginal R^2^ / Conditional R^2^ | 0.008 / 0.086 | | | | | |

Note. Significant results in bold font

**Table S26** Type III ANOVA on the effect of Trial, Group, Profile Type, and their interaction on PEs

| *Predictors* | *Sum of Squares* | *Mean Square* | *df_1_* | *df₂* | *F* | *p* |
| --- | --- | --- | --- | --- | --- | --- |
| Trial | 12.26 | 12.26 | 1 | 13,872.31 | 9.18 | **0.002** |
| Group | 1.51 | 1.51 | 1 | 127.64 | 1.13 | 0.289 |
| Profile Type | 11.55 | 11.55 | 1 | 13,893.68 | 8.65 | **0.003** |
| Trial × Group | 0.23 | 0.23 | 1 | 13,954.29 | 0.17 | 0.680 |
| Trial × Profile Type | 2.19 | 2.19 | 1 | 13,788.83 | 1.64 | 0.200 |
| Group × Profile Type | 6.64 | 6.64 | 1 | 13,956.60 | 4.98 | **0.026** |
| Trial × Group × Profile Type | 0.04 | 0.04 | 1 | 13,954.14 | 0.03 | 0.862 |

Note. *df₁* = numerator degrees of freedom; *df₂* = denominator degrees of freedom; Significant results in bold font

**Table S27** EMMs Contrasts of Group Differences Within Each Profile Type

| *Profile Type* | *Contrast* | *Estimate* | *SE* | *z* | *p* |
| --- | --- | --- | --- | --- | --- |
| BPD | Group BPD - Group CON | -0.003 | 0.055 | -0.054 | 0.957 |
| CON | Group BPD - Group CON | 0.164 | 0.055 | 2.991 | **0.003** |

**Note.** Contrasts reflect differences in predicted values of PEs between Groups within each Profile Type. Degrees-of-freedom method: asymptotic. Df values were not included due to high memory and computation load. Significant results in bold font

**Table S28** Interaction Contrasts of Group × Profile Type on the Linear Effect of Trial (Slopes) on PEs

| *Contrast* | *Estimate* | *SE* | *z* | *p* |
| --- | --- | --- | --- | --- |
| Group BPD Profile Type BPD - Group CON Profile Type BPD | 0.001 | 0.002 | 0.42 | 0.975 |
| Group BPD Profile Type BPD - Group BPD Profile Type CON | 0.003 | 0.003 | 1.055 | 0.717 |
| Group BPD Profile Type BPD - Group CON Profile Type CON | 0.003 | 0.003 | 1.222 | 0.613 |
| Group CON Profile Type BPD - Group BPD Profile Type CON | 0.002 | 0.003 | 0.668 | 0.909 |
| Group CON Profile Type BPD - Group CON Profile Type CON | 0.002 | 0.002 | 0.835 | 0.838 |
| Group BPD Profile Type CON - Group CON Profile Type CON | 0.000 | 0.002 | 0.167 | 0.998 |

Note**.** Contrasts test pairwise differences in the slopes of the Trial effect on PEs across combinations of Group and Profile Type. p-values adjusted using Tukey's method for multiple comparisons. Degrees-of-freedom method: asymptotic. Df values were not included due to high memory and computation load. Significant results in bold font

**Table S29** Estimated Trends (Slopes) for Trial Effects on PE by Group and Profile Type

| Group | Profile Type | Trend (Trial) | SE | LCL | UCL |
| --- | --- | --- | --- | --- | --- |
| BPD | Profile Type BPD | -0.001 | 0.002 | -0.005 | 0.002 |
| CON | Profile Type BPD | -0.002 | 0.002 | -0.005 | 0.001 |
| BPD | Profile Type CON | -0.004 | 0.002 | **-0.007** | **0.000** |
| CON | Profile Type CON | -0.004 | 0.002 | **-0.008** | **-0.001** |

**Note.** Trends represent the estimated linear effect of trial (centered) on PEs for each group × profile type combination. Degrees-of-freedom method: asymptotic. Df values were not included due to high memory and computation load. Confidence level: 95%. LCL = lower confidence limit; UCL = upper confidence limit. Significant results in bold font

## **Computational models**

**Table S30**. Differences in population means of learning rates and initial expectations.

| **Contrast** | **Parameter** | **Posterior Mean Δ** | **Lower 95% SPI** | **Upper 95% SPI** |
| --- | --- | --- | --- | --- |
| CON on CON vs. CON on BPD | alpha_mu | 0.04 | -0.10 | 0.18 |
|  | V0_mu | 0.22 | 0.00 | 0.43 |
| CON on CON vs. CON on BPD | alpha_mu | -0.02 | -0.14 | 0.11 |
|  | V0_mu | -0.18 | -0.40 | 0.06 |
| CON on CON vs. BPD on CON | alpha_mu | 0.02 | -0.12 | 0.16 |
|  | V0_mu | **0.42*** | 0.19 | 0.65 |
| CON on BPD vs. CON on BPD | alpha_mu | -0.03 | -0.15 | 0.09 |
|  | V0_mu | 0.02 | -0.19 | 0.23 |

# **References**

1. Faul F, Erdfelder E, Lang AG, Buchner A. G*Power 3: A flexible statistical power analysis program for the social, behavioral, and biomedical sciences. Behav Res Methods. 2007 May;39(2):175–91. https://doi.org/10.3758/BF03193146

2. Korn CW, La Rosée L, Heekeren HR, Roepke S. Social feedback processing in borderline personality disorder. Psychol Med. 2016 Feb;46(3):575–87. https://doi.org/10.1017/S003329171500207X

3. Frolichs KMM, Rosenblau G, Korn CW. Incorporating social knowledge structures into computational models. Nat Commun. 2022 Oct 20;13(1):6205. https://doi.org/10.1038/s41467-022-33418-2

4. Korn CW, Prehn K, Park SQ, Walter H, Heekeren HR. Positively Biased Processing of Self-Relevant Social Feedback. J Neurosci. 2012 Nov 21;32(47):16832–44. https://doi.org/10.1523/JNEUROSCI.3016-12.2012

5. Carpenter B, Gelman A, Hoffman MD, Lee D, Goodrich B, Betancourt M, et al. *Stan*: A Probabilistic Programming Language. J Stat Softw [Internet]. 2017 [cited 2025 Jul 7];76(1). Available from: http://www.jstatsoft.org/v76/i01/

6. Gabry J, Češnovar R, Johnson A, Bronder S. cmdstanr: R Interface to “CmdStan” [Internet]. 2025. Available from: https://mc-stan.org/cmdstanr/

7. Lee MD, Wagenmakers EJ. Bayesian cognitive modeling: a practical course. Cambridge, United Kingdom New York: Cambridge University Press; 2014. https://doi.org/10.1017/CBO9781139087759

8. Zhang L, Lengersdorff L, Mikus N, Gläscher J, Lamm C. Using reinforcement learning models in social neuroscience: frameworks, pitfalls and suggestions of best practices. Soc Cogn Affect Neurosci. 2020 Jul 30;15(6):695–707. https://doi.org/10.1093/scan/nsaa089

9. Makowski D, Ben-Shachar M, Lüdecke D. bayestestR: Describing Effects and their Uncertainty, Existence and Significance within the Bayesian Framework. J Open Source Softw. 2019 Aug;4(40):1541. https://doi.org/10.21105/joss.01541

10. Vehtari A, Gelman A, Gabry J. Practical Bayesian model evaluation using leave-one-out cross-validation and WAIC. Stat Comput. 2017 Sep;27(5):1413–32. https://doi.org/10.48550/ARXIV.1507.04544
